# Heme metabolism in *Strigomonas culicis*: Implications of H_2_O_2_ resistance induction and symbiont elimination

**DOI:** 10.1016/j.jbc.2024.107692

**Published:** 2024-08-17

**Authors:** Ana Cristina Souza Bombaça, Marcelle Almeida Caminha, Juliana Magalhães Chaves Barbosa, Yasmin Pedra-Rezende, Vitor Ennes-Vidal, Giselle Villa Flor Brunoro, Bráulio Soares Archanjo, Claudia Masini d’Avila, Richard Hemmi Valente, Rubem Figueiredo Sadok Menna-Barreto

**Affiliations:** 1 Laboratório de Biologia Celular, Instituto Oswaldo Cruz, Fundação Oswaldo Cruz, Rio de Janeiro, Brazil; 2 Laboratório de Doenças Parasitárias, Instituto Oswaldo Cruz, Fundação Oswaldo Cruz, Rio de Janeiro, Brazil; 3 Laboratório de Toxinologia, Instituto Oswaldo Cruz, Fundação Oswaldo Cruz, Rio de Janeiro Brazil; 4 Research and Development in Biotechnology, Blau Farmacêutica S/A, São Paulo, Brazil; 5 Divisão de Metrologia de Materiais, Instituto Nacional de Metrologia, Qualidade e Tecnologia (Inmetro), Duque de Caxias, Brazil

**Keywords:** *Strigomonas culicis*, protozoan, hydrogen peroxide, symbiosis, heme, iron, mass spectrometry (MS), parallel reaction monitoring

## Abstract

Monoxenous trypanosomatid *Strigomonas culicis* harbors an endosymbiotic bacterium, which enables the protozoa to survive without heme supplementation. The impact of H_2_O_2_ resistance and symbiont elimination on intracellular heme and Fe^2+^ availability was analyzed through a comparison of WT strain with both WT H_2_O_2_-resistant (WTR) and aposymbiotic (Apo) protozoa. The relative quantification of the heme biosynthetic pathway through label-free parallel reaction monitoring targeted mass spectrometry revealed that H_2_O_2_ resistance does not influence the abundance of tryptic peptides. However, the Apo strain showed increased coproporphyrinogen III oxidase and ferrochelatase levels. A putative ferrous iron transporter, homologous to *L*IT1 and *Tc*IT from *Leishmania major* and *Trypanosoma cruzi*, was identified for the first time. Label-free parallel reaction monitoring targeted mass spectrometry also showed that *S. culicis* Iron Transporter (*Sc*IT) increased 1.6- and 16.4-fold in WTR and Apo strains compared to WT. Accordingly, antibody-mediated blockage of *Sc*IT decreased by 28.0% and 40.0% intracellular Fe^2+^concentration in both WTR and Apo strains, whereas no effect was detected in WT. In a heme-depleted medium, adding 10 μM hemin decreased *Sc*IT transcript levels in Apo, whereas 10 μM PPIX, the substrate of ferrochelatase, increased intracellular Fe^2+^ concentration and ferric iron reduction. Overall, the data suggest mechanisms dependent on *de novo* heme synthesis (and its substrates) in the Apo strain to overcome reduced heme availability. Given the importance of heme and Fe^2+^ as cofactors in metabolic pathways, including oxidative phosphorylation and antioxidant systems, this study provides novel mechanistic insights associated with H_2_O_2_ resistance in *S. culicis*.

The Trypanosomatidae family (Euglenozoa: Kinetoplastea) is well recognized for sheltering the causative agents of important human illnesses, such as Chagas disease, sleeping sickness, and leishmaniasis; however, most of its members are nonpathogenic insect protozoa (1). The Strigomonadinae subfamily comprises species of genera *Angomonas*, *Strigomonas*, and *Kentomonas*, which are midgut-dwelling insect protozoa bearing an endosymbiotic β-proteobacterium known as *Candidatus* Kinetoplastibacterium spp. (Alcaligenaceae) (2, 3). Since endosymbiosis has long been considered a central phenomenon in the origin of organelles, the study of trypanosomatids and their symbionts is of great interest in characterizing genetic, metabolic, and evolutionary aspects of this process (4). The endosymbiotic relationship in the *Strigomonadinae* subfamily is characterized by tight control of coordinated symbiont division and host cell cycle, allowing each protozoan to carry only one bacterium (5, 6, 7). Symbiont provides the host cell with several vital nutrients, such as essential amino acids (8), vitamins (9) and heme (10), conferring physiological advantages within nutritionally challenging environments.

Heme (iron-protoporphyrin IX) is an iron-containing molecule acting in many biological processes (11). Most trypanosomatids do not present the heme biosynthetic pathway, requiring the addition of hemoglobin, hematin, or hemin in the medium for successful *in vitro* cultivation (12). However, symbiont-harboring trypanosomatids (SHT) can grow in a chemically defined medium without any source of heme, whereas aposymbiotic strains (‘cured’ by antibiotic treatment) cannot (13, 14). Alves et al. (10) detected all genes required for heme synthesis in SHTs in a cooperation between host trypanosomatid and symbiont genomes. The nuclear genome of SHTs presents genes for glutamyl-tRNA synthetase (GltX), which is essential for protein synthesis, as well as the genes for coproporphyrinogen III oxidase (CPOX), protoporphyrinogen oxidase (PPOX), and ferrochelatase (FeCH). In addition, the genomes of their symbionts bear all the genes to complement heme synthesis using the C-5 pathway, including glutamyl-tRNA reductase (HemA), glutamate-1-semialdehyde 2,1-aminomutase (GsaM) to make δ-aminolevulinic acid, and the enzymes to produce coproporphyrinogen III from δ-aminolevulinic acid, including δ-aminolevulinic acid dehydratase (ALAD), porphobilinogen deaminase (PBGD), uroporphyrinogen III synthase (UROS), uroporphyrinogen III decarboxylase (UROD), and oxygen-independent coproporphyrinogen III oxidase (HemN).

The biological utilization of iron depends on its transitional property, which can adopt either the reduced ferrous (Fe^2+^) or oxidized ferric (Fe^3+^) states to carry out diverse redox conversions within the cell. The ability of iron to readily undergo oxidation/reduction cycles also leads to its inherent toxicity and is involved in the generation of various free radicals *via* the Fenton reaction, such as the hydroxyl radical (OH^•^) (15); therefore, iron acquisition and storage systems must be tightly regulated. *Leishmania* Iron Transporter 1 (*L*IT1) (16) and *Trypanosoma cruzi* Iron Transporter (*Tc*IT) (17) belong to the family of zinc/iron-regulated proteins (ZIP) of metal transporters, which contains the ferrous iron transporter IRT1 from *Arabidopsis thaliana* (18). ZIP proteins are predicted to share a similar membrane topology, with five to eight transmembrane domains and the amino- and carboxy-terminal ends located on the extracellular side of the plasma membrane. In addition, the length of different ZIP family members ranges from 227 to 476 amino acid residues due to a variable cytoplasmic region between transmembrane domains III and IV (16, 19, 20). Iron-depleted medium induces *L*IT1 expression in *Leishmania amazonensis* and triggers the differentiation of promastigotes (extracellular insect-stage parasite) into amastigotes (intracellular mammalian-stage parasite), increasing Fe-superoxide dismutase (FeSOD) activity and hydrogen peroxide (H_2_O_2_) production (21). Interestingly, in addition to the differentiation of *T. cruzi* epimastigotes (extracellular insect-stage parasite) into trypomastigotes (extracellular mammalian-stage parasite), *Tc*IT overexpression increases mitochondrial O_2_ consumption, ATP content, and H_2_O_2_ production (17).

As an iron-protoporphyrin, heme plays multifaceted roles in cellular physiology, acting as a cofactor in essential enzymatic reactions and as a regulator of redox balance through its participation in electron transport systems and redox reactions. Moreover, as a component of peroxidases, heme directly influences the cellular defense against reactive oxygen species (ROS) by consuming H_2_O_2_ (22). In previous work, our group induced H_2_O_2_ resistance in the *Strigomonas culicis* WT strain through protozoa incubation with increasing H_2_O_2_ concentrations. This process correlates with enhanced mitochondrial activity, characterized by increased O_2_ consumption and elevated ATP production. Additionally, there is a significantly higher abundance and activity of antioxidant heme-proteins, such as ascorbate peroxidase (ASCPx), in the H_2_O_2_-resistant WT (WTR) strain. Conversely, symbiont elimination in aposymbiotic (Apo) strain leads to impaired mitochondrial functionality and decreased abundance of heme proteins associated with oxidative phosphorylation (OXPHOS), such as cytochrome *c*, cytochrome *c* oxidase, and cytochrome *c* reductase (23, 24). Despite the large data set showing the genomic cooperation in SHTs (25), the understanding of heme availability in the WT and Apo strains is limited. Additionally, there is scarce knowledge concerning the mechanisms governing its synthesis and uptake in the WTR strain. This work evaluated the abundance of the heme biosynthetic pathway through a label-free parallel reaction monitoring targeted mass spectrometry (PRM-MS) and determined the intracellular heme content in WT, WTR, and Apo strains. Moreover, a membrane protein homologous to ferrous iron transporters *L*IT1 and *Tc*IT was characterized for the first time in a monoxenous trypanosomatid, suggesting that ferrous iron transport systems may be conserved in the Trypanosomatidae family.

## Results

### WTR strain has increased intracellular heme concentration, while the Apo protozoa show a decrease in its level

**Figure 1:**
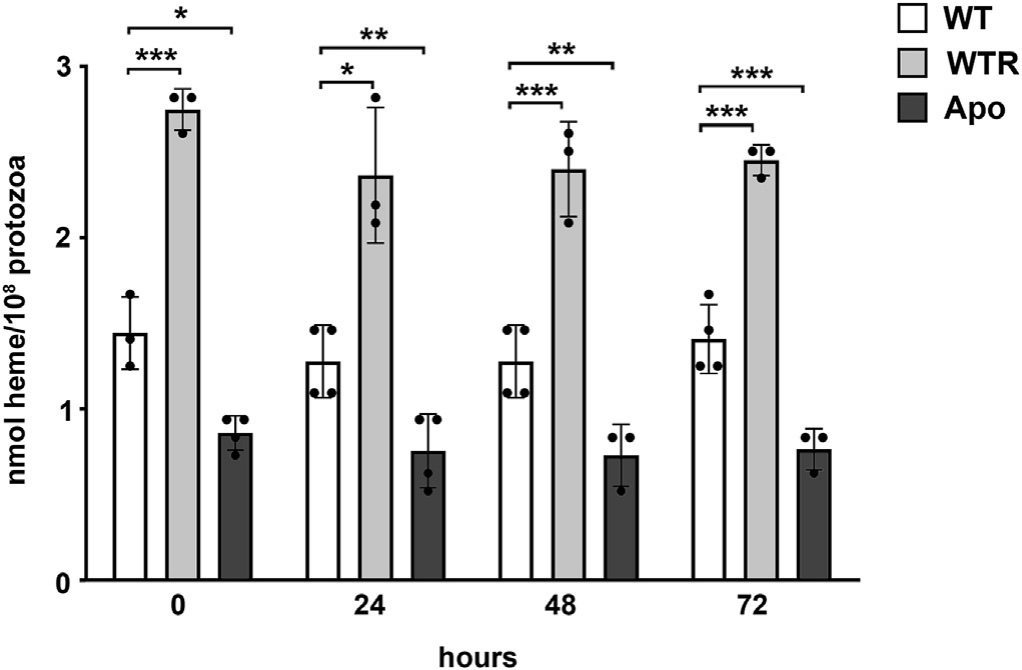
**Intracellular heme concentration was modulated in WTR and Apo strains.** Determination of intracellular heme concentration along the initial 72 h of the WT, WTR, and Apo strains grown in SM at 28 °C. Data are represented as mean ± SD of at least three independent experiments. Significant *p*-values were obtained by one-way ANOVA test followed by Tukey's post-test (∗*p* ≤ 0.05; ∗∗*p* ≤ 0.03; ∗∗∗*p* ≤ 0.01).

To determine whether the H_2_O_2_ resistance induction and symbiont elimination in *S. culicis* influence intracellular heme availability, its content was assayed during protozoa growth (0, 24, 48, and 72 h) in standard medium (SM), which was composed by liver infusion and tryptose (LIT) media supplemented with 10% heat-inactivated fetal bovine serum (FBS) and 15 μM bovine hemin (26). At all analyzed times, WTR presented 1.7-fold higher heme levels than WT, whereas Apo had intracellular heme concentration decreased by up to 45%. Nevertheless, intracellular heme concentration remained stable in WT, WTR, and Apo strains without significant time-dependent change (Fig. 1).

### WTR strain does not exhibit any alteration in the heme biosynthetic pathway, whereas Apo protozoa has an increased abundance of CPOX and FeCH

**Figure 2:**
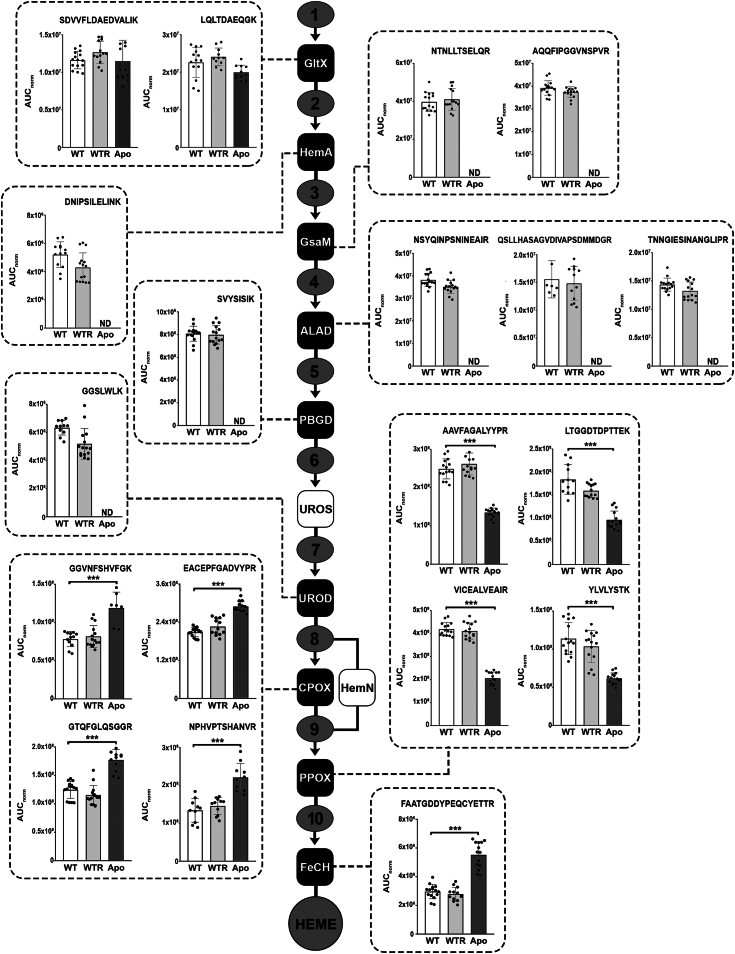
**Heme biosynthetic pathway was not modulated in the WTR strain, whereas coproporphyrinogen III oxidase and ferrochelatase were increased in Apo protozoa.** Tryptic peptides from proteins of the heme biosynthetic pathway were monitored in WT, WTR, and Apo strains grown in SM for 72 h at 28 °C. *Dark rectangles* represent proteins monitored by label-free PRM-MS, while *white rectangles* represent proteins whose peptides were not detected by the DDA analysis in any strain. Compounds: L-glutamate (1); L-glutamyl-tRNA (2); glutamate-1-semialdehyde (3); δ−aminolevulinic acid (4); porphobilinogen (5); hydroxymethylbilane (6); uroporphyrinogen III (7); coproporphyrinogen III (8); protoporphyrinogen IX (9); protoporphyrin IX (10). Data are represented as mean ± SD of five independent experiments in at least technical duplicate. Significant *p*-values were obtained by one-way ANOVA test followed by Dunnett's post-test (∗∗∗*p* ≤ 0.01). ALAD, δ−aminolevulinic acid dehydratase; CPOX, coproporphyrinogen III oxidase; FeCH, Ferrochelatase; GsaM, glutamate-1-semialdehyde 2,1-aminomutase; GltX, Glutamyl-tRNA synthetase; HemA, Glutamyl-tRNA reductase; HemN, oxygen-independent coproporphyrinogen III oxidase; ND, not detected; PBGD, porphobilinogen deaminase; PPOX, protoporphyrinogen oxidase; UROD, uroporphyrinogen III decarboxylase; UROS, uroporphyrinogen III synthase.

**Figure S1:**
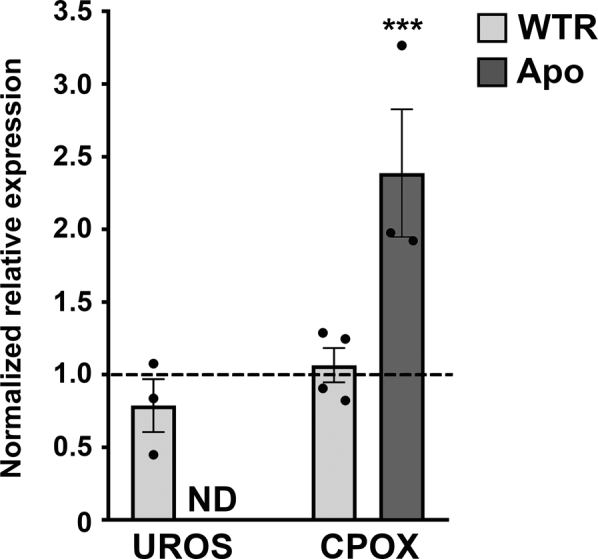


Considering that *S. culicis* exhibits heme autotrophy, its biosynthetic pathway was analyzed using a mass spectrometry approach in protozoa grown for 72 h in SM. Firstly, a list of 69 tryptic peptides was generated by an *in silico* approach, clustering 13 proteins responsible for heme synthesis (three to eight peptides for each protein). Through data-dependent acquisition (DDA) analysis, 27.5% of them were successfully identified in at least one of the three strains, and their spectral data were used to build a library in Skyline software (Table S1). Thus, a final panel of 19 tryptic peptides belonging to nine proteins of the heme biosynthetic pathway was ultimately monitored for relative quantification through label-free PRM-MS (Table S2). As shown in Figure 2, it was possible to quantify, through the normalized area under the curve (AUC_norm_; considering a coefficient variation ≤20%), the abundance of GltX, HemA, GsaM, ALAD, PBGD, UROD, CPOX, PPOX, and FeCH in both WT and WTR. In contrast, only GltX, CPOX, PPOX, and FeCH were monitored in the Apo. The AUC_norm_ analysis revealed that WTR did not show significant alterations in the abundance of any analyzed peptides. In contrast, although the quantity of GltX was not modulated in Apo, the last three enzymes of the heme biosynthetic pathway were changed. On average, peptides from CPOX and FeCH were 1.6- and 1.9-fold increased, whereas PPOX abundance decreased in the range of 45.8 to 50.0% in Apo, compared to the WT (Fig. 2). As it was impossible to monitor the abundance of UROS, since its peptides were not detected by DDA analysis, transcript levels coding for this protein were evaluated. There was no difference in UROS gene expression between WT and WTR; as expected, its transcripts were undetected in the Apo. Confirming the correlation between real-time quantitative PCR (qPCR) data and protein abundance concerning the heme biosynthetic pathway, the CPOX gene transcript levels were not modulated in WTR and increased 2.4-fold in Apo (Fig. S1).

### *S. culicis* has an ATP-dependent heme uptake, which is increased in WTR strain

**Figure 3:**
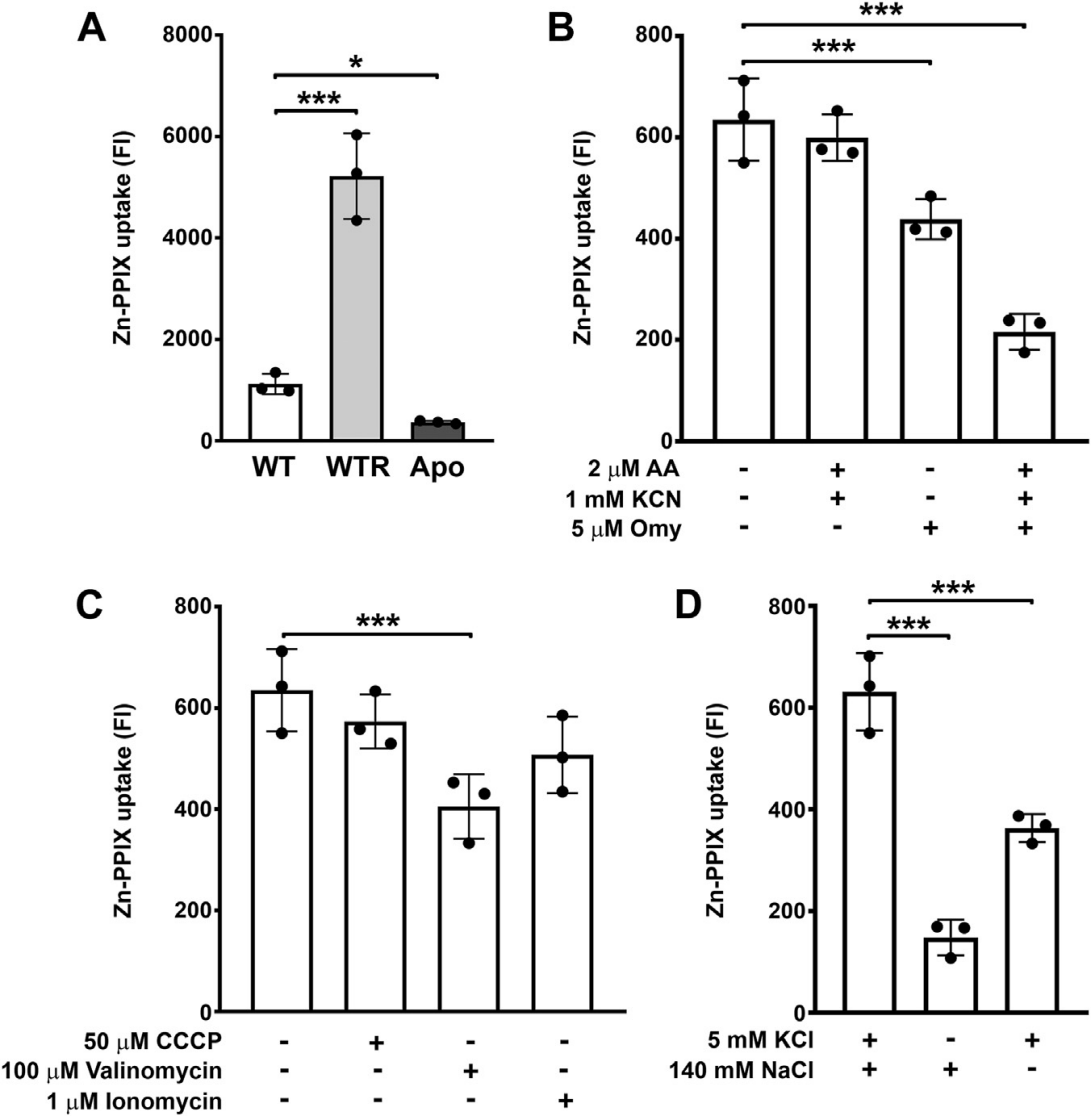
**Heme uptake was increased in the WTR strain.***A*, the uptake of heme analog Zn-PPIX was measured in WT, WTR, and Apo strains grown in SM for 72 h at 28 °C. *B* and *C*, WT protozoa were pre-incubated with (*B*) ATP depleters (5 μM Omy, 2 μM AA, and 1 mM KCN) or (*C*) ionophores (50 μM CCCP, 100 μM valinomycin, and 1 μM ionomycin) in combination to Zn-PPIX to evaluate whether heme uptake is an ATP-dependent process. *D*, WT protozoa were also incubated in MES buffer containing or not KCl and/or NaCl to evaluate whether heme uptake requires monovalent ions. Data are represented as mean ± SD of three independent experiments. Significant *p*-values were obtained by one-way ANOVA test followed by Dunnett's post-test (∗*p* ≤ 0.05; ∗∗∗*p* ≤ 0.01).

To account for whether intracellular heme concentration is increased in WTR due to its uptake (since the abundance of heme biosynthetic pathway was similar in WT), zinc(II)-protoporphyrin IX (Zn-PPIX) uptake was measured in protozoa grown for 72 h in SM. As shown in Figure 3*A*, the uptake of the fluorescent heme analog Zn-PPIX increased 4.7-fold in WTR and decreased 67.2% in Apo in comparison to WT (Fig. 3*A*). To evaluate whether heme uptake in *S. culicis* may be an ATP-dependent process, the fluorescence of Zn-PPIX was measured in WT protozoa pretreated with ATP depleters and in the presence of 50 μM carbonyl cyanide m-chlorophenyl hydrazone (CCCP), 100 μM valinomycin, and 1 μM ionomycin, which dissipate the H^+^, K^+^, and Ca^2+^ gradients, respectively. The decrease in ATP levels derived from WT treatment with 5 μM oligomycin A (Omy) alone or combined with 2 μM antimycin A (AA) + 1 mM KCN impaired Zn-PPIX uptake up to 66.0% (Fig. 3*B*). Although uptake of Zn-PPIX was not significantly altered in the presence of CCCP and ionomycin, it decreased by 35.0% with valinomycin (Fig. 3*C*). Confirming this result, WT exhibited a decrease in 76.5% and 42.5% in Zn-PPIX uptake in the absence of K^+^ and Na^+^ ions in 4-morpholineethanesulfonic acid (MES) buffer, respectively (Fig. 3*D*).

**Figure S2:**
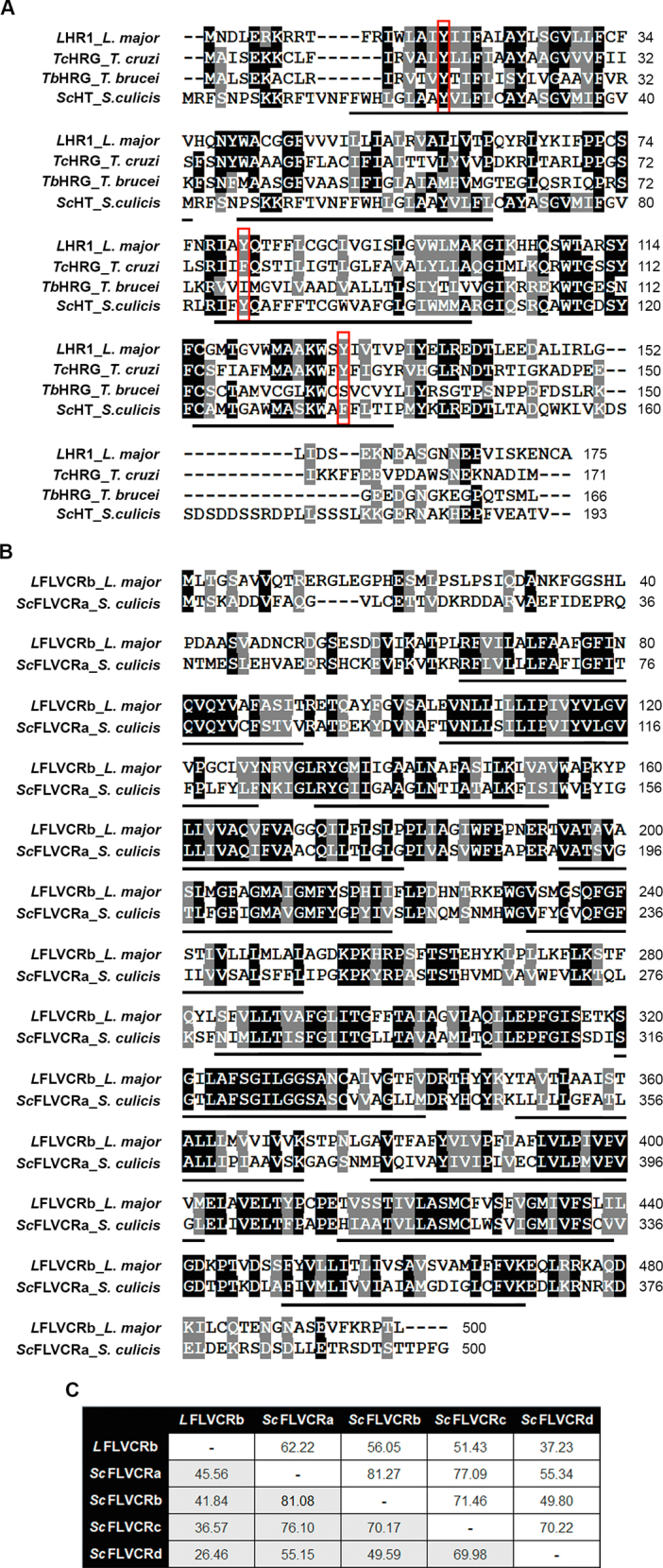


Sequences of heme transporters, previously described in other trypanosomatids, were used as templates to search for homologous proteins in the *S. culicis* database. BLAST homology searches revealed a putative protein with 193 amino acids and 22.2 kDa, assigned as an "uncharacterized protein", similar in molecular mass and transmembrane segments to proteins belonging to the HRG family, such as *Leishmania* Heme Response-1 (*L*HR1), *T. cruzi* Heme Response Gene (*Tc*HRG), and *Trypanosoma brucei* Heme Response Gene(*Tb*HRG). The protein (GenBank accession no. ATMH01004537), hereafter referred to as *S. culicis* Heme Transporter (*Sc*HT), shares 43.0% identity and 57.9% similarity with *L*HR1, 33.9% identity and 50.5% similarity with *Tc*HRG, and 28.0% identity and 44.9% similarity with *Tb*HRG. The predicted membrane topology of *Sc*HT suggests the presence of four putative transmembrane segments with the amino- and carboxy-terminal ends located on the protozoa cytoplasm. Besides their similarities in primary structure and predicted topology, tyrosine residues essential for heme transport in *L*HR1 (Tyr-18 and Tyr-80) (27) are conserved in the *S. culicis* protein, which correspond to Tyr-24 and Tyr-86. In addition, Tyr-129, another relevant tyrosine in *L*HR1 (27), is substituted by phenylalanine in *Sc*HT (Phe-135) (Fig. S2*A*). A putative protein with 500 amino acids and 54.5 kDa, assigned as “*major facilitator superfamily (MFS) profile domain-containing protein*”, was also identified in the *S. culicis* database. The protein (GenBank accession no. ATMH01005666), hereafter referred to as *S. culicis* FLVCRa (*Sc*FLVCRa), shares 45.6% identity and 62.2% similarity with *Leishmania* FLVCRb (*L*FLVCRb), a member of the MSF superfamily of membrane transporters. Topological prediction suggests the presence of 12 putative transmembrane segments with the amino- and carboxy-terminal ends located on the protozoa cytoplasm (Fig. S2*B*). *Sc*FLVCRb, *Sc*FLVCRc, and *Sc*FLVCRd (GenBank accession no. ATMH01000240, ATMH01001865, and ATMH01001634, respectively) were also identified; however, these proteins possess decreasing degrees of identity and similarity with *L*FLVCRb and *Sc*FLVCRa (Fig. S2*C*). Although the data presented suggest that *S. culicis* can uptake heme from the extracellular environment, further analyses must be conducted to confirm the presence and functionality of *Sc*HT and *Sc*FLVCRa.

### Apo strain exhibits enhanced growth and heme synthesis after the addition of the FeCH substrate

**Figure 4:**
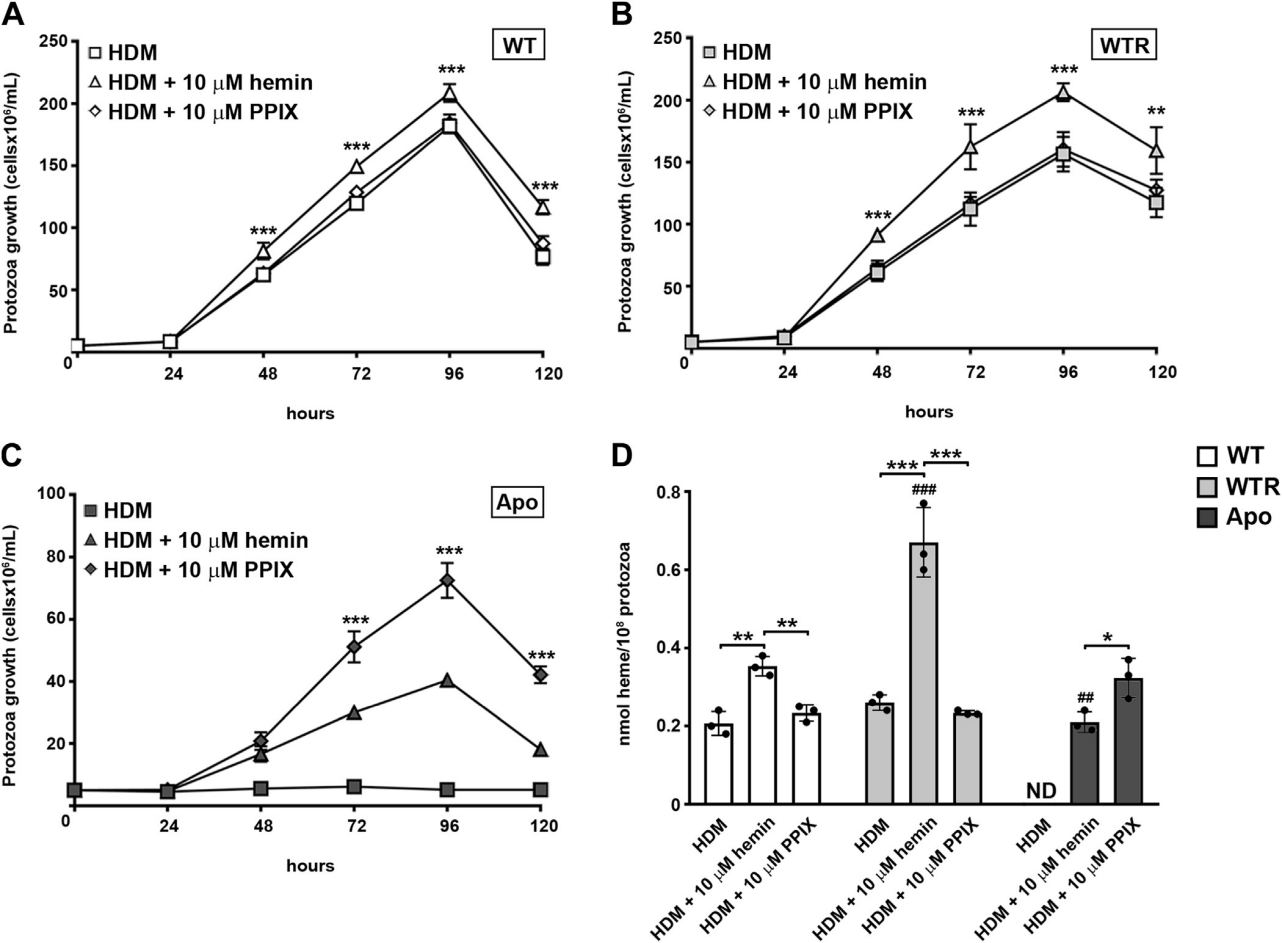
**FeCH substrate supported the heme biosynthetic pathway and improved the growth of the Apo strain.** Growth curves of (*A*) WT, (*B*) WTR, and (*C*) Apo strains cultured in HDM, HDM + 10 μM hemin, or HDM + 10 μM PPIX at 28 °C for up to 120 h. Data are represented as mean ± SD of at least three independent experiments. Significant *p*-values were obtained comparing HDM + 10 μM hemin with HDM + 10 μM PPIX by one-way ANOVA test followed by Tukey's post-test (∗∗*p* ≤ 0.03; ∗∗∗*p* ≤ 0.01). *D*, intracellular heme concentration of protozoa was maintained in HDM alone or with 10 μM hemin or 10 μM PPIX for 72 h at 28 °C. Data are represented as mean ± SD of three independent experiments. Significant *p*-values were obtained by one-way ANOVA test followed by Tukey's post-test for the indicated comparisons (∗*p* ≤ 0.05; ∗∗*p* ≤ 0.03; ∗∗∗*p* ≤ 0.01) or in comparison to the WT strain grown in HDM + 10 μM hemin (^##^*p* ≤ 0.03; ^###^*p* ≤ 0.01). ND, not detected.

To determine the relevance of uptake and biosynthetic pathway to intracellular heme availability, protozoa were grown for up to 120 h in heme-depleted medium (HDM), HDM + 10 μM hemin, or HDM + 10 μM protoporphyrin IX (PPIX), the substrate of FeCH. WT and WTR were able to proliferate in HDM. Also, after 48 h of growth, both strains reached higher levels when cultured with HDM + hemin instead of PPIX (Fig. 4, *A* and *B*). HDM could not sustain Apo growth; however, adding hemin or PPIX allowed the protozoa to grow similarly to that detected for the WT, albeit at a lower density. Interestingly, Apo cultured with HDM + PPIX had a more expressive growth after 72 h than those maintained with HDM + hemin (Fig. 4*C*). The growth of WT with HDM or HDM + PPIX maintained intracellular heme concentration at similar levels (0.21 ± 0.03 and 0.23 ± 0.02 nmol, respectively) and increased by 1.7-fold only when the protozoa were cultured with HDM + hemin. Similarly, intracellular heme concentration in the WTR increased 2.6-fold when the protozoa were grown in HDM + hemin (0.26 ± 0.02–0.67 ± 0.09 nmol). Although intracellular heme concentration was not detected in the Apo strain grown in HDM, the addition of both hemin and PPIX enabled its determination. Intracellular heme concentration was 1.5-fold increased in Apo strain grown in HDM + PPIX. The significant differences concerning intracellular heme concentration, detected in WT x WTR and WT x Apo comparisons, disappeared when protozoa were grown in HDM + PPIX instead of hemin (Fig. 4*D*).

### WTR and Apo strains present an increase in gene expression and protein abundance of the ferrous iron transporter *Sc*IT

**Figure 5:**
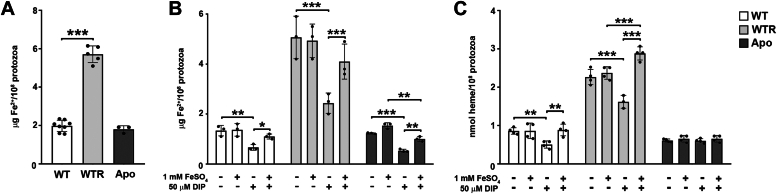
**Intracellular Fe**^**2+**^**concentration was increased in the WTR strain and not modulated in Apo protozoa.***A*, determination of intracellular Fe^2+^ concentration of the WT, WTR, and Apo strains grown in SM for 72 h at 28 °C. Additionally, intracellular (*B*) Fe^2+^and (*C*) heme concentrations were analyzed in protozoa cultured in SM with or without the addition of 1 mM FeSO_4_, 50 μM DIP, or 50 μM DIP + 1 mM FeSO_4_ for 72 h at 28 °C. DIP was used to deplete iron from the culture medium to produce a condition of low iron availability. Data are represented as mean ± SD of at least three independent experiments. Significant *p*-values were obtained by one-way ANOVA test followed by Tukey's or Dunnett's post-tests for the indicated comparisons (∗*p* ≤ 0.05; ∗∗*p* ≤ 0.03; ∗∗∗*p* ≤ 0.01).

Since the final step in the heme biosynthetic pathway is incorporating ferrous iron into the protoporphyrin ring by FeCH, its intracellular concentration was assessed in protozoa grown for 72 h in SM. WTR had 3.0-fold higher intracellular Fe^2+^ concentration than WT, whereas no significant difference was observed between this strain and Apo (Fig. 5*A*). To evaluate the effect of an iron-decreased environment on intracellular heme availability, protozoa were grown for 72 h in SM plus 50 μM 2,2′-dipyridyl (DIP), a metal ion chelating ligand (28). As observed in Figure 5*B*, the presence of DIP decreased intracellular Fe^2+^ concentration in the three strains. In the WT, treatment with DIP decreased 47.7% intracellular Fe^2+^ concentration compared to untreated control; this impairment in WTR and Apo was 52.4 and 55.3%, respectively. This phenotype was at least partially protected by the combined addition of 50 μM DIP + 1 mM FeSO_4_; intracellular Fe^2+^ levels increased by 1.5- to 1.8-fold across the strains (Fig. 5*B*). The presence of DIP decreased by 41.1% and 28.6% intracellular heme concentration in WT and WTR, respectively, compared to the untreated control. Once again, the combination of DIP + FeSO_4_ improved the analysis, increasing in up to 1.7-fold the detection of intracellular heme. Interestingly, incubation with DIP did not cause any effect on the intracellular heme levels in Apo (Fig. 5*C*).

**Figure 6:**
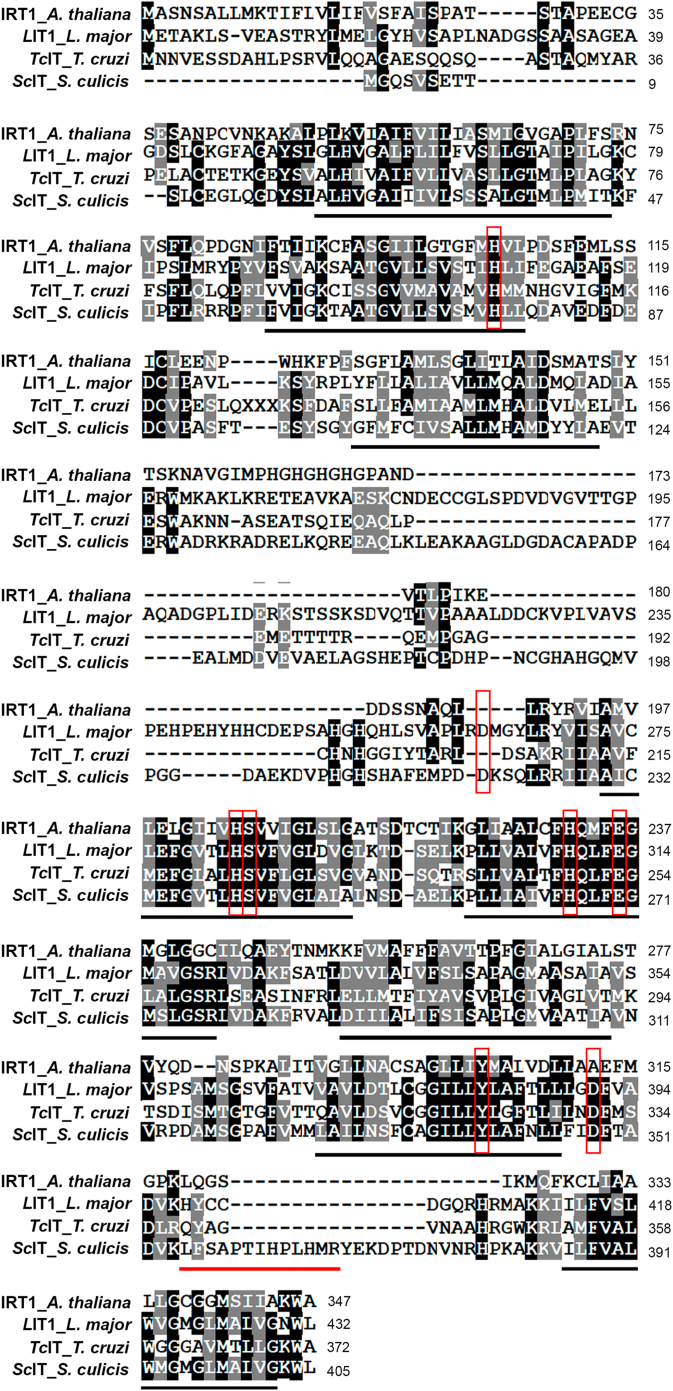
***Sc*IT, a membrane protein homologous to *L*IT1 and *Tc*IT, is encoded by the *Strigomonas culicis* genome.** Amino acid sequence alignments showing the proteins *Sc*IT from *S. culicis*, *L*IT1 from *L. major*, *Tc*IT from *T. cruzi*, and IRT1 from *A. thaliana*. Identical residues are highlighted in *black*, and similar residues in *gray*. Predicted transmembrane segments are underlined in *black*, while the peptide (LFSAPTIHPLHMR), monitored by label-free PRM-MS, is underlined in *red*. Conserved residues critical for the Fe^2+^ transport function of *L*IT1 and IRT1 are boxed in *red*.

**Figure 7:**
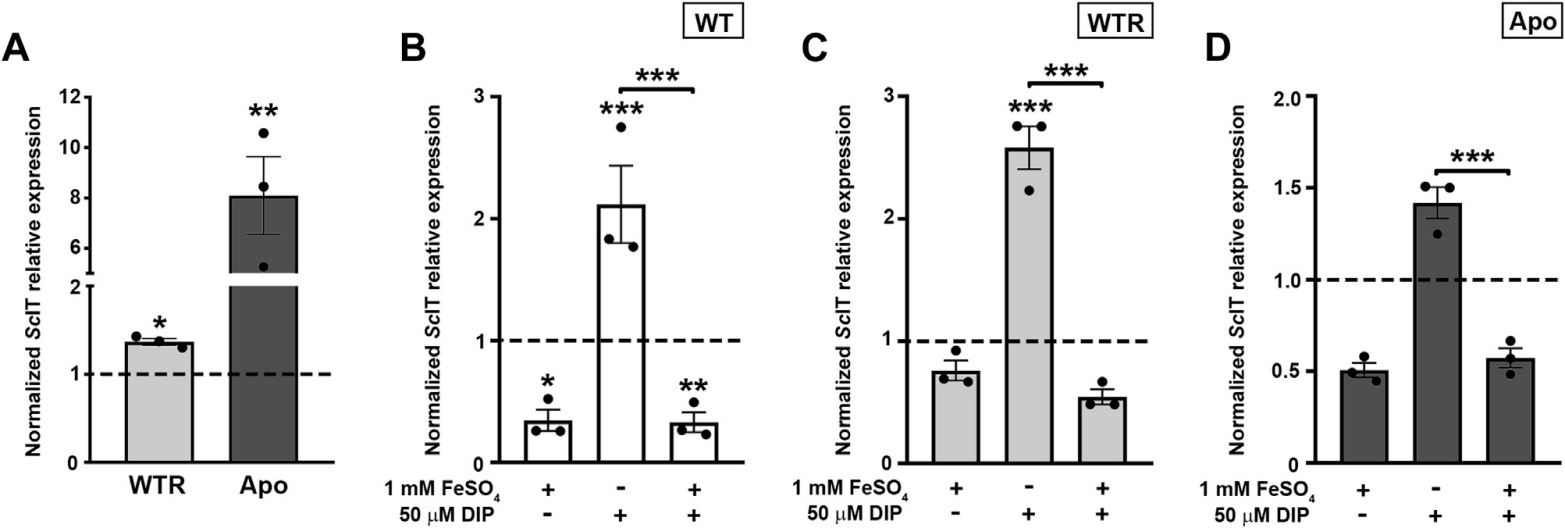
***Sc*IT gene expression was increased in WTR and Apo strains.***A*, gene expression analysis of *Sc*IT in WTR and Apo strains grown in SM for 72 h at 28 °C. Data were normalized using the transcript levels of WT strain (*dashed black line*). Additionally, gene expression analysis was performed in (*B*) WT, (*C*) WTR, and (*D*) Apo strains cultured in SM plus 1 mM FeSO_4_, 50 μM DIP, or 50 μM DIP + 1 mM FeSO_4_ for 72 h at 28 °C. Data were normalized using the transcript levels of WT, WTR, and Apo strains maintained in SM (*dashed black line*). Actin and 69 kDa paraflagellar rod protein were used as endogenous controls in all experimental design. Data are represented as sampling distribution of the mean ± SEM of three independent experiments in at least technical duplicate. Significant *p*-values were obtained by one-way ANOVA test followed by Dunnett's post-test (∗*p* ≤ 0.05; ∗∗*p* ≤ 0.03; ∗∗∗*p* ≤ 0.01).

To evaluate whether the machinery for ferrous iron uptake was present in *S. culicis*, a search was carried out for proteins homologous to *L*IT1, *Tc*IT, and IRT1. BLAST homology searches revealed the putative protein assigned as "*Solute carrier family 39 (Zinc transporter), member 1/2/3*" (GenBank accession no. ATMH01009756) in the *S. culicis* database, hereafter referred to as *S. culicis* Iron Transporter (*Sc*IT). *Sc*IT sequence alignment had 46.4% identity and 58.0% similarity with *L*IT1, 34.6% identity and 55.0% similarity with *Tc*IT, and 29.4% identity and 42.5% similarity with IRT1 (Fig. 6). *Sc*IT is a 405 amino acid protein of 44.2 kDa predicted to contain eight transmembrane segments and amino- and carboxy-terminal ends located on the extracellular side of the plasma membrane. *Sc*IT has His-76, His-240, Ser-241, His-266, and Glu-270, which are amino acid residues conserved among ZIP family members and essential for *L*IT1 biological activities (29). *Sc*IT also possesses Tyr-339, Asp-220, and Asp-348 corresponding to Tyr-382, Asp-263, and Asp-391 in *L*IT1 (29) (Fig. 6). Analysis of *Sc*IT gene expression in protozoa grown in SM showed that its transcript was 1.4- and 8.1-fold increased in WTR and Apo, compared to WT (Fig. 7*A*). The incubation of WT, WTR, and Apo with DIP increased the *Sc*IT gene expression by 2.1-, 2.6-, and 1.4-fold, respectively, compared to the untreated control. Noticeably, a negative modulation in *Sc*IT transcription, in response to the addition of FeSO_4_, was only noted in WT (Fig. 7, *B*–*D*).

**Figure 8:**
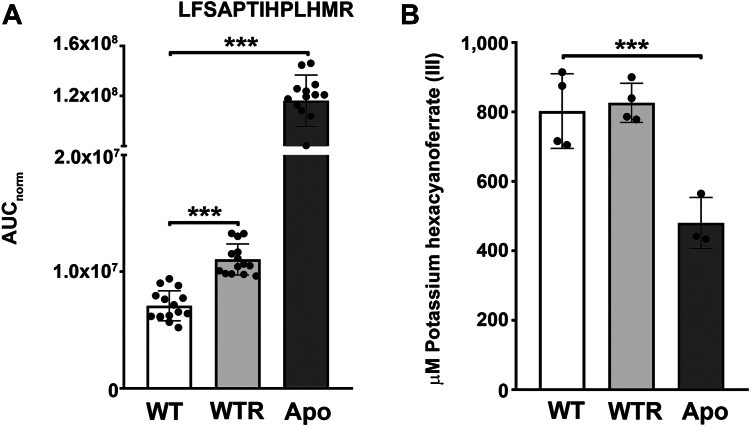
***Sc*IT abundance was increased in WTR and Apo strains.***A*, the tryptic peptide from *Sc*IT (LFSAPTIHPLHMR) was monitored by label-free PRM-MS and detected in WT, WTR, and Apo strains grown in SM for 72 h at 28 °C. Data are represented as mean ± SD of five independent experiments in at least technical duplicate. Significant *p*-values were obtained by the Kruskal–Wallis test followed by Dunn's post-test (∗∗∗*p* ≤ 0.01). Additionally, (*B*) ferric reductase activity of protozoa cultured in SM for 72 h at 28 °C was measured through the reduction of the ferric into the ferrous form of K_3_[Fe(CN)_6_]. Data are represented as mean ± SD of at least three independent experiments. Significant *p*-values were obtained by one-way ANOVA test followed by Dunnett's post-test for the indicated comparisons (∗∗∗*p* ≤ 0.01).

The abundance of *Sc*IT peptides was also analyzed by mass spectrometry in WT, WTR, and Apo strains grown for 72 h in SM. Although three tryptic peptides from *Sc*IT (DPTDNVNR, DVPHGHSHAFEMPDDK, and LFSAPTIHPLHMR) (Table S1) have been selected through an *in silico* approach, only one was detected by DDA and quantified by the label-free PRM-MS. Results of AUC_norm_ demonstrated that WTR and Apo had the abundance of LFSAPTIHPLHMR 1.6- and 16.4-fold increased in relation to the WT, respectively (Fig. 8*A*). Considering that insoluble ferric iron must be converted to soluble ferrous iron before being transported across membranes, a ferric reductase assay utilizing the membrane-impermeable compound K_3_[Fe(CN)_6_] was also performed. As shown in Figure 8*B*, no difference was detected in the extracellular concentration of K_3_[Fe(CN)_6_] comparing WT and WTR (802.7 ± 107.6 and 826.2 ± 56.3 μM, respectively), whereas in Apo, a decrease of 40.2% was observed in comparison to WT (Fig. 8*B*).

**Figure 9:**
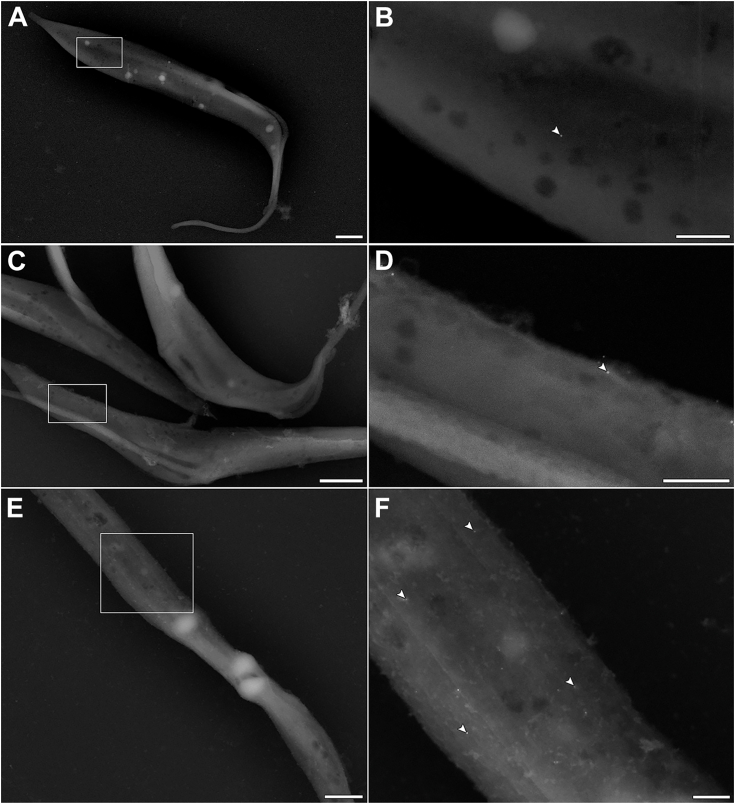
**Ultrastructural analysis indicates the presence of *Sc*IT on the surface of *Strigomonas culicis*.***Sc*IT was detected on the surface of the three *S. culicis* strains by combining SEM with immunogold labeling. (*A* and *B*) WT, (*C* and *D*) WTR, and (*E* and *F*) Apo strains were incubated in MES buffer containing the primary rabbit anti-*Sc*IT polyclonal antibody (dilution 1:50) prior to 2% paraformaldehyde fixation and incubation with secondary goat anti-rabbit IgG-gold 10 nm antibody (dilution 1:75). *B*, *D*, and *F*, micrographs of the areas enclosed by the *white rectangle* were enlarged to visualize the gold particles better. *White* arrowheads indicate isolated *gold* particles on the protozoa surface. All images were obtained *via* BSE, using a CBS detector, at an accelerating voltage of 18 kV. Bars represent 1 μm (*A*, *C* and *E*) and 250 nm (*B*, *D*, and *F*).

**Figure 10:**
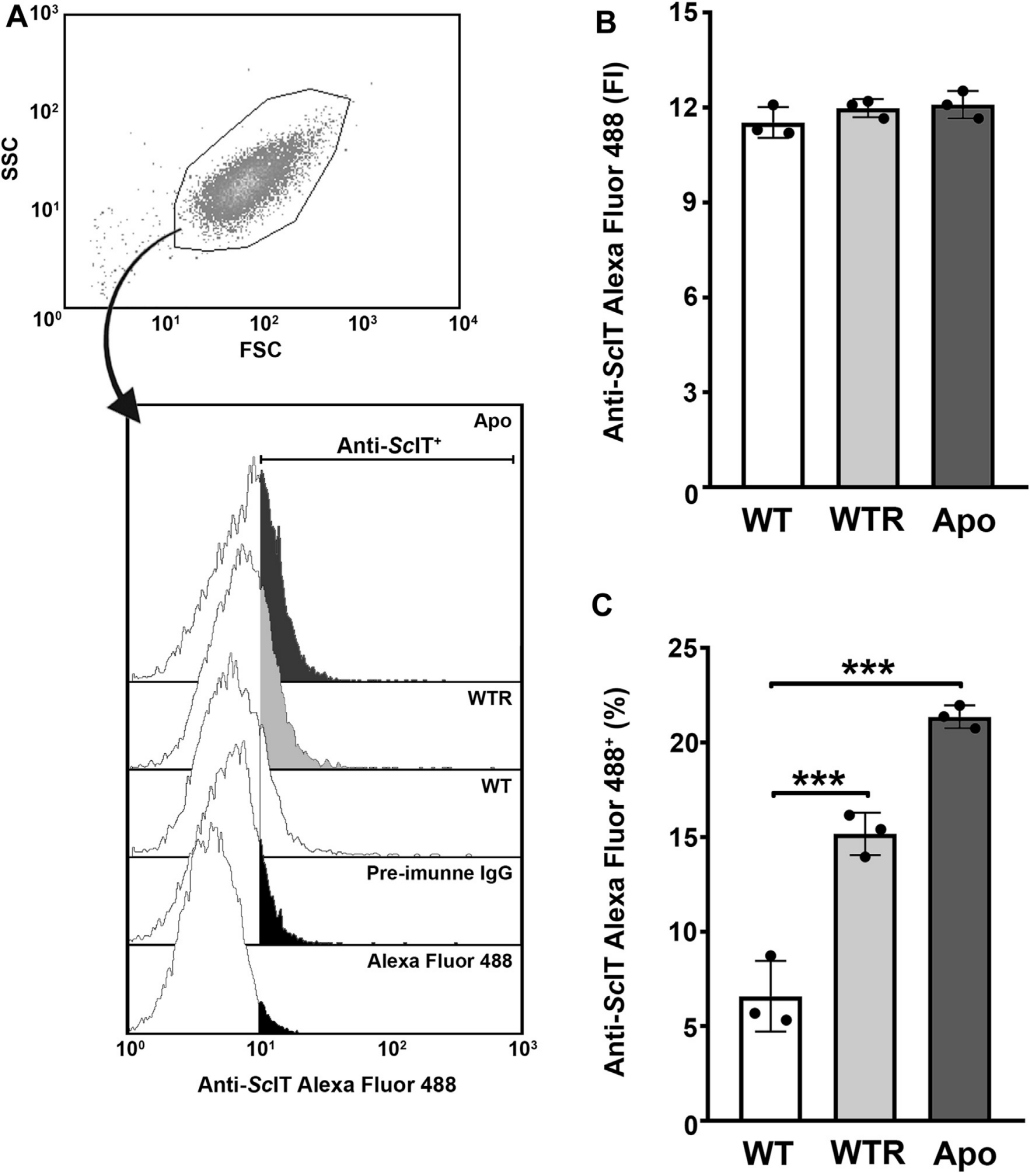
**Detection of *Sc*IT, through protozoa labeling with anti-*Sc*IT antibody, also increased in WTR and Apo strains.** WT, WTR, and Apo strains grown in SM for 72 h at 28 °C were labeled with primary rabbit anti-*Sc*IT polyclonal antibody (dilution 1:125) and secondary goat anti-rabbit Alexa Fluor 488 antibody (dilution: 1:2000) for flow cytometry analysis. *A*, representative gating strategy and histograms of anti-*Sc*IT AlexaFluor 488. *B*, fluorescence intensity of anti-*Sc*IT AlexaFluor 488 and (*C*) percentage of protozoa labeled with anti-*Sc*IT AlexaFluor 488. Data are represented as mean ± SD of three independent experiments. Significant *p*-values were obtained by one-way ANOVA test followed by Dunnett's post-test (∗∗∗*p* ≤ 0.01).

**Figure 11:**
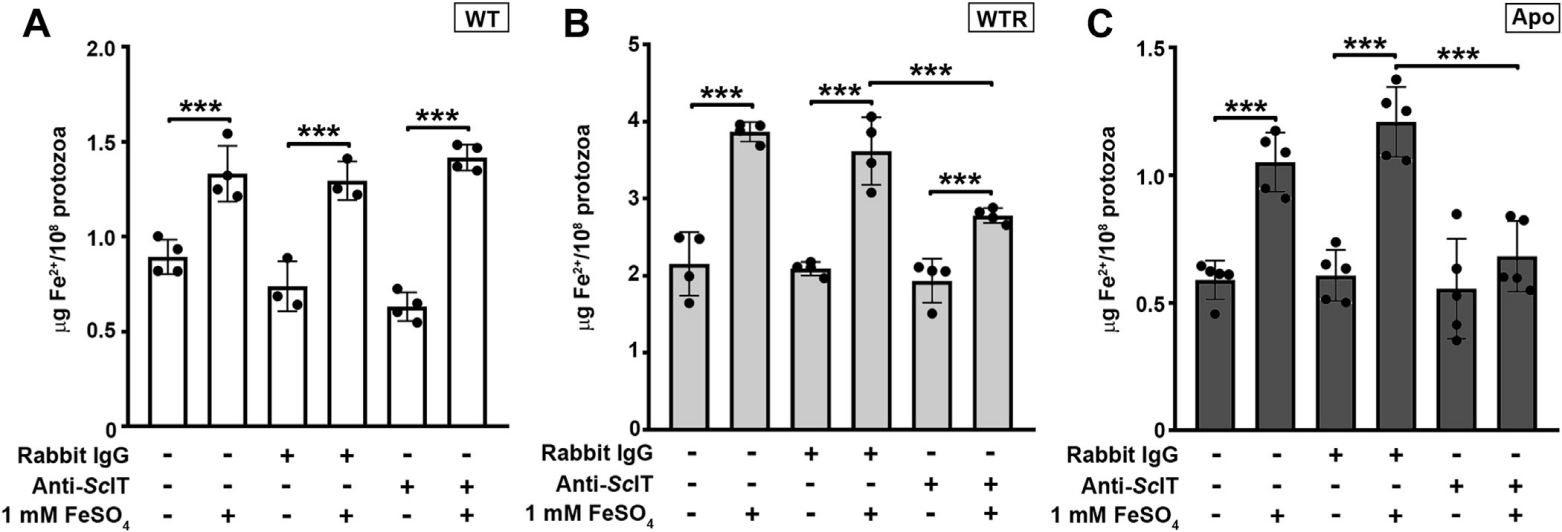
**Anti-*Sc*IT antibody decreased Fe**^**2+**^**uptake in WTR and Apo strains.** Determination of intracellular Fe^2+^ concentration in (*A*) WT, (*B*) WTR, and (*C*) Apo strains grown in SM for 72 h at 28 °C. Analysis was performed in MES buffer supplemented with 10% FBS containing primary rabbit anti-*Sc*IT polyclonal antibody (dilution 1:50) or pre-immune rabbit IgG (dilution 1:50) to conduct an antibody-mediated blockage. Eventually, 1 mM FeSO_4_ was added to evaluate Fe^2+^ uptake. Data are represented as mean ± SD of at least three independent experiments. Significant *p*-values were obtained by one-way ANOVA test followed by Tukey's post-test (∗∗∗*p* ≤ 0.01).

**Figure S3:**
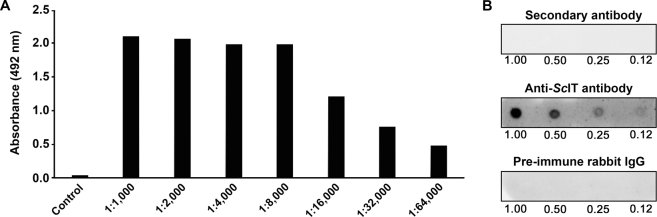


**Figure S4:**
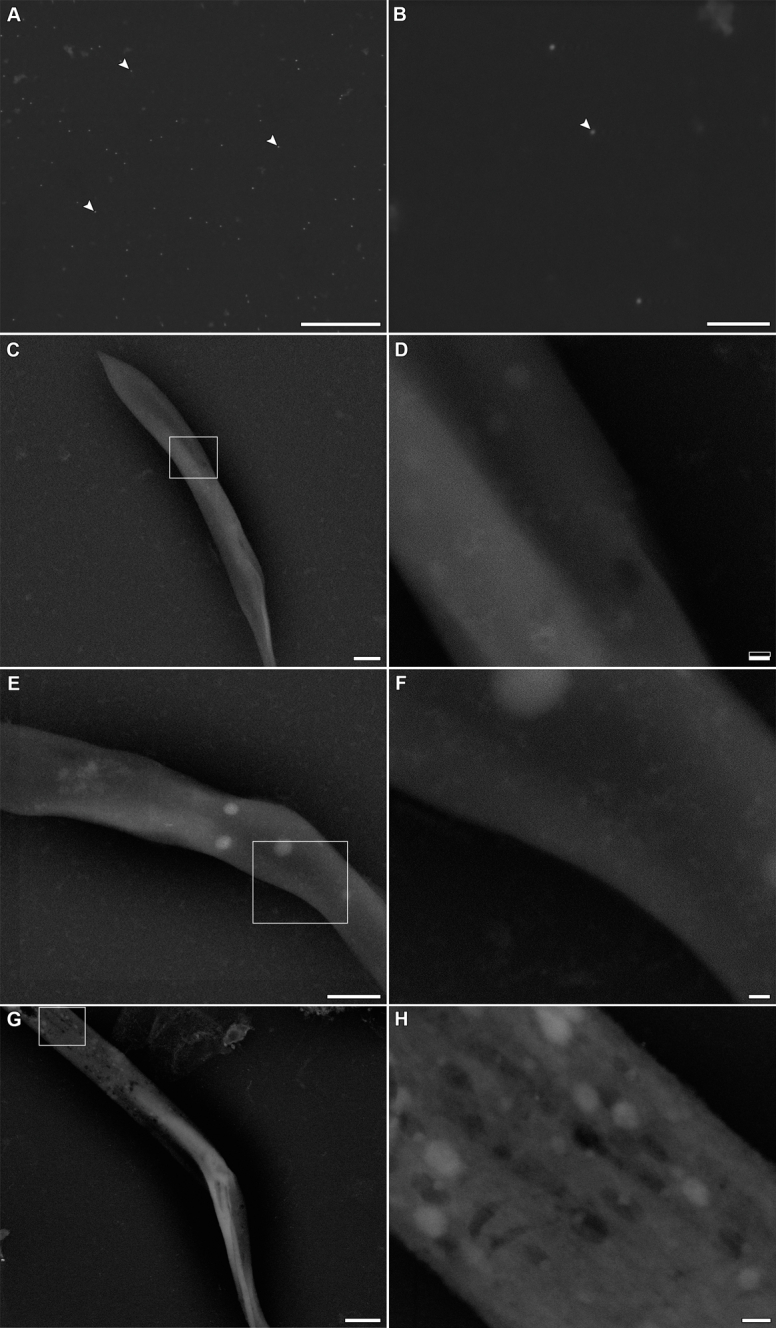


To better characterize the ferrous iron transporter in *S. culicis*, a polyclonal antibody was produced against an extracellular portion of the *Sc*IT amino-terminal region (MGQSVSETTSLCEGLQGDYS), following the strategy described by Huynh et al. (16). Although the anti-*Sc*IT antibody failed to detect the endogenous full-length *Sc*IT by immunoblot, it efficiently identified the protozoa's extracellular epitope (Fig. S3). Thus, anti-*Sc*IT was used for detection of *Sc*IT by combining scanning electron microscopy (SEM) with immunogold labeling. As shown in immune gold SEM micrographs of protozoa grown for 72 h in SM, gold particles were found in the cell surface of WT, WTR, and Apo strains (Fig. 9). The nonspecific labeling was determined through protozoa incubation with pre-immune ("naive") rabbit IgG and showed to be negligible (Fig. S4). Confirming the mass spectrometry data, flow cytometry analysis of labeled protozoa revealed no significant differences in the fluorescence intensity between the three strains (median values of 11.5 ± 0.5, 12.0 ± 0.3, and 12.1 ± 0.4 for WT, WTR, and Apo, respectively) (Fig. 10, *A* and *B*). Concerning the percentage of labeled protozoa, both WTR and Apo showed an increase of 2.4- and 3.2-fold comparatively to WT, respectively (Fig. 10*C*). To assess the contribution of *Sc*IT to intracellular Fe^2+^ availability, protozoa were incubated with an anti-*Sc*IT antibody, to block the ferrous iron transporter through steric hindrance, and the Fe^2+^ concentration was determined. As shown in Figure 11*A*, incubation of WT with rabbit IgG or anti-*Sc*IT antibody did not interfere with intracellular Fe^2+^ concentration in the presence of 1 mM FeSO_4_ (Fig. 11*A*). In contrast, while no effect was detected with rabbit IgG, protozoa incubation with anti-*Sc*IT antibody led to a partial decrease in Fe^2+^ uptake by WTR and completely abolished it in Apo. In the presence of FeSO_4_, intracellular Fe^2+^ concentration was 28.0% and 40.0% decreased by anti-*Sc*IT antibody in WTR and Apo, respectively (Fig. 11, *B* and *C*).

### The addition of FeCH substrate induces an increase in *Sc*IT abundance and Fe^2+^ uptake in the Apo strain

**Figure 12:**
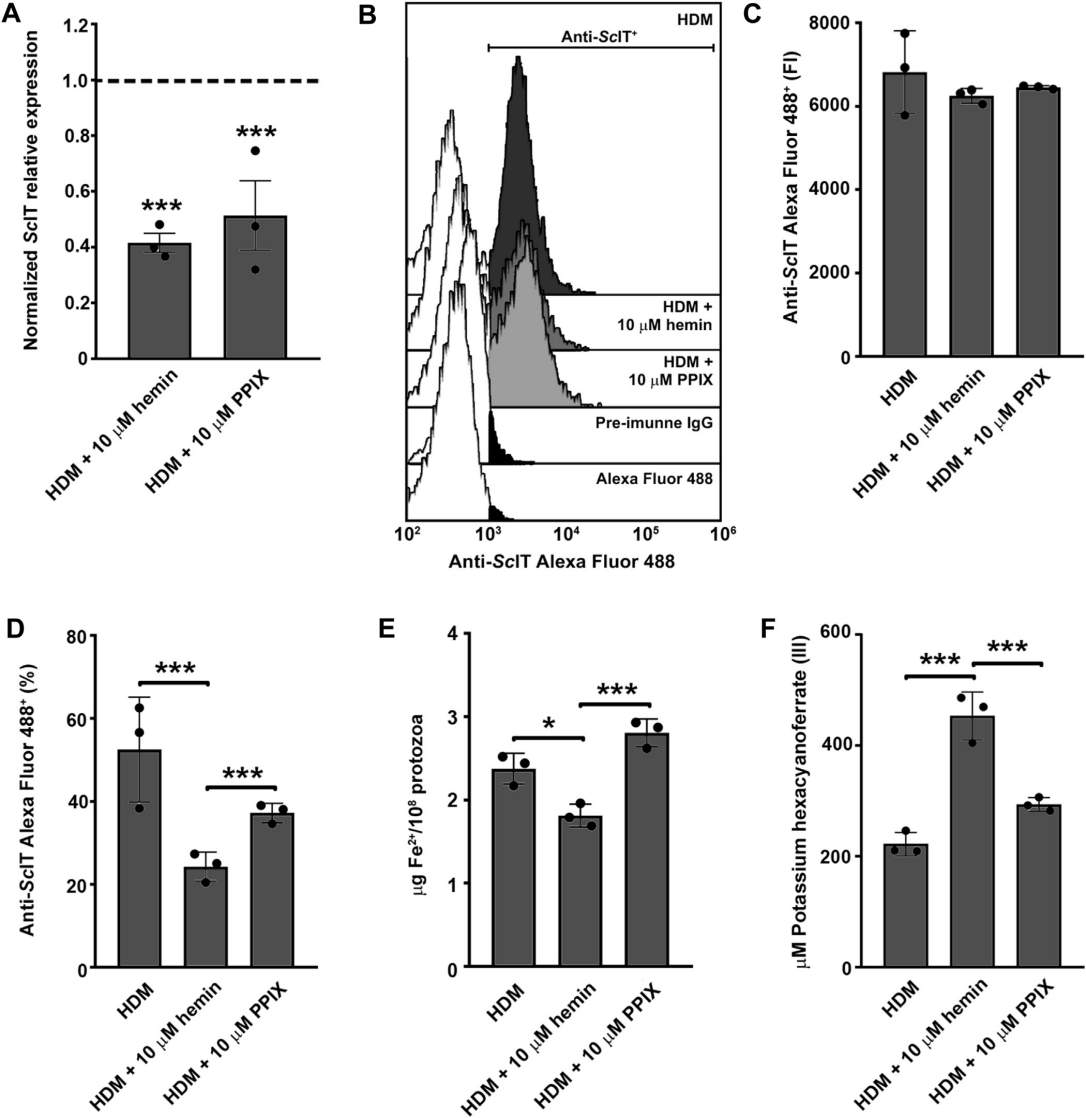
***Sc*IT abundance enhanced in response to the heme-decreased environment and FeCH substrate in the Apo strain.***A*, gene expression analysis of *Sc*IT in Apo strain cultured in HDM + 10 μM hemin or HDM +10 μM PPIX for 72 h at 28 °C. Data were normalized using the transcript levels of the Apo strain cultured in HDM (*dashed black line*). Actin and 69 kDa paraflagellar rod protein were used as endogenous controls. Data are represented as sampling distribution of the mean ± SEM of three independent experiments in at least technical duplicate. Significant *p*-values were obtained by one-way ANOVA test followed by Dunnett's post-test (∗∗∗*p* ≤ 0.01). *B*–*D*, flow cytometry analysis of Apo strain cultured in HDM alone or with 10 μM hemin or 10 μM PPIX for 72 h at 28 °C. Protozoa were labeled with primary rabbit anti-*Sc*IT polyclonal antibody (dilution 1:125) and secondary goat anti-rabbit Alexa Fluor 488 antibody (dilution 1:2000). *B*, representative histograms of anti-*Sc*IT AlexaFluor 488. *C*, fluorescence intensity of anti-*Sc*IT AlexaFluor 488 and (*D*) percentage of protozoa labeled with anti-*Sc*IT AlexaFluor 488. Determination of (*E*) intracellular Fe^2+^ concentration and (*F*) ferric reductase activity of Apo strain cultured in HDM alone or with 10 μM hemin or 10 μM PPIX for 72 h at 28 °C. Data are represented as mean ± SD of three independent experiments. Significant *p*-values were obtained by one-way ANOVA test followed by Tukey's or Dunnett's post-test (∗*p* ≤ 0.05; ∗∗∗*p* ≤ 0.01).

*Sc*IT could be upregulated in the Apo strain to enable heme synthesis, so its expression was determined in protozoa grown for 72 h in HDM, HDM + 10 μM hemin, or HDM +10 μM PPIX. Adding hemin or PPIX to HDM decreased 58.4% and 48.6% *Sc*IT transcript levels in the Apo strain (Fig. 12*A*). Flow cytometry analysis using anti-*Sc*IT antibody did not show significant differences in the fluorescence intensity among all three experimental conditions; however, a decrease of 53.8% was detected in the percentage of labeled protozoa after adding hemin to HDM (Fig. 12, *B*–*D*). Compared to HDM, intracellular Fe^2+^ content decreased by 23.7% in the protozoa submitted to HDM + hemin, whereas no significant changes were detected in the HDM + PPIX (Fig. 12*E*). Accordingly, the extracellular concentration of K_3_[Fe(CN)_6_] was 2.0-fold higher after Apo growth in HDM + hemin (Fig. 12*F*).

## Discussion

The Trypanosomatidae family is predominantly composed of monoxenous species, which were considered, for many years, as less relevant than its pathogenic relatives. This scenario has been changing, and these species attracted attention due to their high diversity, ability to adapt to different environmental conditions, and impact on their host insect populations (30, 31). In SHTs, the symbiotic relationship confers to monoxenous protozoa, the ability to synthesize heme and grow without any source of this iron-protoporphyrin (13, 14, 32). Here, a targeted mass spectrometry approach was employed to monitor the abundance of most proteins of the heme biosynthetic pathway in *S. culicis*. Gene expression in trypanosomatids is almost exclusively controlled by posttranscriptional mechanisms, making DNA- or RNA-based analyses unreliable for protein identification. In this scenario, proteomic data can provide a broader picture of protein presence in the protozoa (33), and targeted mass spectrometry is an effective tool for monitoring sets of low-abundance peptides with high reproducibility (34). Despite the efforts of our group, the previous proteomic studies comparing *S. culicis* strains could not detect proteins involved in heme synthesis (24, 35). Here, employing the label-free PRM-MS approach, most of the proteins involved in this pathway were identified and quantified. Although some proteins were monitored by only one peptide, all peptides detected, regardless of the protein and the pathway stage, exhibited the same abundance profile, increasing the reliability of the label-free PRM-MS data. It is well-known that the symbiont genome codifies most proteins responsible for this pathway; however, some overlap functions with the protozoa's genes. The lack of detection of any peptides from HemN and the symbiont genome-encoded isoforms of GltX and FeCH suggest that only copies codified by the *S. culicis* genome can perform a function during heme synthesis.

Peptides of all monitored proteins of the heme biosynthetic pathway were identified in the WT and WTR strains. However, H_2_O_2_ resistance induction did not modulate this pathway in either strain. On the other hand, label-free PRM-MS analysis revealed increased peptide abundance of CPOX and FeCH in the Apo strain. Together with PPOX, these proteins are responsible for *de novo* heme synthesis. CPOX, PPOX, and FeCH were acquired from a γ-proteobacterium through lateral gene transfer and are encoded by some monoxenous trypanosomatids and *Leishmania* (22). In L. *major*, the presence of these three proteins enables intracellular amastigotes to utilize heme precursors scavenged from macrophages to fulfill its requirements despite the parasite not producing an earlier precursor of the heme biosynthetic pathway. However, these proteins are not required to generate typical lesions in a murine model of cutaneous leishmaniasis, and knockout promastigotes from *Lm*FeCH have a normal development inside the insect vector (36). The increased abundance of CPOX and FeCH in the Apo strain appears to be an adaptive mechanism to circumvent the deleterious effects of low heme uptake and intracellular concentration due to the absence of symbiont. Despite these efforts, coproporphyrinogen III, the substrate of CPOX, is not available extracellularly in the insect midgut or in the culture medium, which prevents heme synthesis.

Despite the results obtained by the label-free PRM-MS approach, a significant difference was observed in the intracellular heme concentration comparing WT x WTR and WT x Apo strains, indicating a heme uptake system in *S. culicis*. Currently, two direct heme uptake systems have been described in *Leishmania*. *L*HR1 and *L*FLVCRb promote heme import into the cytosol and are essential for the parasite, given that null mutants were not viable. Also, in both cases, single-knockout parasites cannot replicate intracellularly in macrophages and show a severe defect in the development of cutaneous lesions in mice (37, 38). Although *L*HR1 and *L*FLVCRb share the heme importer at the plasma membrane, the overlap function between both is incomplete, suggesting that *L*HR1 and *L*FLVCRb could play other essential tasks in *Leishmania*. Further analyses must confirm whether *Sc*HT and *Sc*FLVCRa act separately or together to scavenge heme from the extracellular environment in *S. culicis*. The predicted membrane topologies of *Sc*HT and *Sc*FLVCRa were consistent with the proposed for *L*HR1, *L*FLVCRb, *Tc*HRG, and *Tb*HRG (37, 38, 39, 40). *Sc*HT has preserved the amino acid residues previously described as critical for heme import (27, 37). Interestingly, Zn-PPIX uptake in *S. culicis* decreased in the presence of ATP depleters and ionophores, suggesting that heme uptake is a carrier-mediated secondary active transport process that requires the presence of Na^+^ and/or K^+^ ions. This phenotype was similar to the one that Cabello-Donayre and colleagues (41) observed in a study characterizing the kinetic properties of *L*FLVCRb.

Although WTR has increased intracellular heme and Fe^2+^ concentrations, protozoa growth in an iron-decreased environment only partially impaired the heme content (at levels similar to those detected for the WT strain). Also, the growth of WT and WTR strains in HDM or HDM + PPIX disrupted the significant difference in intracellular heme concentration, previously detected in protozoa cultured in SM or HDM + hemin. Altogether, these data confirm the PRM-MS results, showing no modulation of the heme biosynthetic pathway in response to H_2_O_2_ resistance induction, and suggest that the increase of heme content in WTR strain is due to its improved uptake. Despite the increase in uptake, it is not possible to ignore the role of the heme degradation pathway in the maintenance of its intracellular levels. Heme catabolism is catalyzed by the enzyme heme oxygenase, resulting in the formation of biliverdin, carbon monoxide, and Fe^2+^ (42); however, the presence of this protein in trypanosomatids is still unclear (43). Although the heme oxygenase gene is absent in the *T. cruzi* genome and no enzymatic activity has been recorded, the occurrence of intermediates of heme catabolism (α-meso-hydroxyheme, verdoheme, and biliverdin) has been described in epimastigotes (44, 45). Even in *Plasmodium falciparum*, in which the expression and activity of a heme oxygenase-like protein have been demonstrated, the canonical heme degradation pathway does not occur (46, 47). Thus, further analyses must be conducted to elucidate which processes benefit from the increased heme uptake in the WTR strain, especially when it appears not to be essential for the protozoa viability. Furthermore, efforts should be directed to elucidate whether the heme degradation pathway (canonical or non-canonical) is present in *S. culicis*, and if it is altered in response to H_2_O_2_ resistance induction.

Trypanosomatids require iron for crucial processes; however, the source of this molecule and its acquisition differ according to the protozoan species and the host they inhabit (48). This work characterized a direct ferrous iron incorporation pathway in *S. culicis*, the first identified in monoxenous trypanosomatids. *Sc*IT is homologous to *L*IT1 and *Tc*IT from *L. major* and *T. cruzi*, respectively (16, 17). Indeed, analysis of predicted amino acid sequences showed common features between *Sc*IT, *L*IT1, and *Tc*IT, such as the number of transmembrane domains, localization of amino- and carboxy-terminal ends, and essential components for heavy-metal binding sites (29). Also, data using an anti-*Sc*IT antibody to block the ferrous iron transporter demonstrate that the activity of this protein significantly contributes to the maintenance of intracellular Fe^2+^ content in the WTR and Apo strains. The data obtained with anti-*Sc*IT antibody also suggest the existence of other metabolic pathways involved in iron acquisition in *S. culicis*, especially in the WT strain. *T. brucei* acquires iron from its hosts through the uptake of transferrin (49), a glycosylated Fe^3+^-binding protein found in blood plasma and other tissue fluids, whose primary function is to transport iron to all cells. Also, in *T. cruzi*, transferrin receptors are present in the epimastigote form (which can uptake transferrin *via* the endocytic pathway), indicating the use of this molecule as an iron source (50, 51). The uptake of transferrin by *S. culicis* may be one factor that allows the maintenance of intracellular Fe^2+^ content in the WT strain and partially contributes to its overall concentration in the WTR. It is important to note that WT and WTR strains can infect the midgut of hematophagous insects, such as *Aedes aegypti* (24), where they encounter elevated concentrations of transferrin; this *in vivo* infection does not occur with the Apo strain (23).

Regardless of the mechanism, heme and Fe^2+^ must get into the protozoa, where they are required for the assembly and activity of several heme-proteins and Fe-S clusters. ASCPx enhancement was related to the ROS- and reactive nitrogen species (RNS)-resistance in *Leishmania donovani* and *Leishmania braziliensis* (52, 53). The ROS-inducible ASCPx of *L. amazonensis* was also essential for the survival of all parasite life cycle stages, *in vitro* infection of murine macrophages, and the development of cutaneous lesions in mice (54). In the same manner, knockout amastigotes from *L*IT1 failed to replicate within macrophages and were avirulent, being unable to generate lesions in highly susceptible mice, even after extensive periods of time (16). *L*IT1 overexpression induces FeSOD activity in *L. amazonensis*, suggesting that intracellular iron availability, maintained by the ferrous iron transporter, is essential in generating active antioxidant enzymes (21). Similarly, overexpression of *Tc*IT in *T. cruzi* results in increased activity of FeSOD and decreased production of H_2_O_2_ (17). The increased intracellular heme and Fe^2+^ concentrations detected in WTR may be responsible for the previously described increment in the abundance and activity of FeSOD and ASCPx (24), but it must be further investigated. Curiously, during the infection of *A. aegypti* females with WTR strains, there is a downregulation of inducible-catalase and an increase in H_2_O_2_ production by midgut epithelial cells (55). Thus, the increased antioxidant defenses, especially ASCPx, would favor protozoa survival in this environment (23).

Previous data showed that the Apo strain had a decreased abundance of heme-proteins in the mitochondria, such as cytochrome *c* reductase and cytochrome *c* oxidase, which was reflected in the lower activity of mitochondrial complexes II-III and IV, and compromised ATP production (23, 24). The fact that mitochondrion is one of the most relevant heme-protein-containing organelle (56) could explain the compensatory mechanism previously suggested by our group (24), based on the exacerbation of glucose consumption and pentose phosphate pathway to supply ATP and reducing molecules in the Apo strain. *Angomonas deanei* symbiont-free protozoa also showed impaired mitochondrial O_2_ consumption and ATP synthesis by OXPHOS (57). This suggests that bacterium elimination may decrease intracellular heme content and mitochondrial heme-protein activity of other SHTs. Koreny and colleagues (58) demonstrated that the trypanosomatid *Phytomonas serpens* can survive without heme, and only the FeCH gene was found in its genome; thus, this parasite lacks cytochromes, peroxidases, and other heme-proteins. However, its ATP production is based on glycolysis since these parasites live in carbohydrate-rich environments (58, 59).

The analysis performed by Silva and colleagues (60) demonstrated that *Kentomonas sorsogonicus*, a SHT, is unable to grow in the absence of an exogenous heme source in its culture medium. In this case, its endosymbiont retained only the genes coding for HemN and FeCH. The authors raised the question that, in this scenario, the impaired heme biosynthetic pathway may be advantageous for *K. sorsogonicus*, allowing its growth solely with heme provided by its invertebrate host (as is the case of almost all trypanosomatids). This strategy may avoid the regulation of heme synthesis and intracellular accumulation of cytotoxic molecules, such as free iron. Previous work from our group showed that the Apo strain had increased H_2_O_2_ production in a mechanism non-responsive to pretreatment with antioxidants (23). Due to the absence of the CPOX substrate in the invertebrate host and the culture medium, heme synthesis does not occur in the Apo strain. Thus, it is possible to assume that part of the Fe^2+^ uptake by *Sc*IT can remain free in the cytoplasm, contributing to the increase in ROS generation. It is important to note that this hypothesis is supported by the rise in *Sc*IT and intracellular Fe^2+^ concentrations in protozoa cultured with HDM or HDM + PPIX, the substrate of FeCH, and by the decrease of both in protozoa grown in HDM + hemin. The increased H_2_O_2_ production in the Apo strain is accompanied by the low abundance of two FeSOD isoforms (23, 24). This suggests that the intracellular Fe^2+^ concentration in the Apo strain may not contribute to the generation of active antioxidant enzymes, and raises the question of whether symbiont may participate in protein synthesis and enzymatic activity.

**Figure 13:**
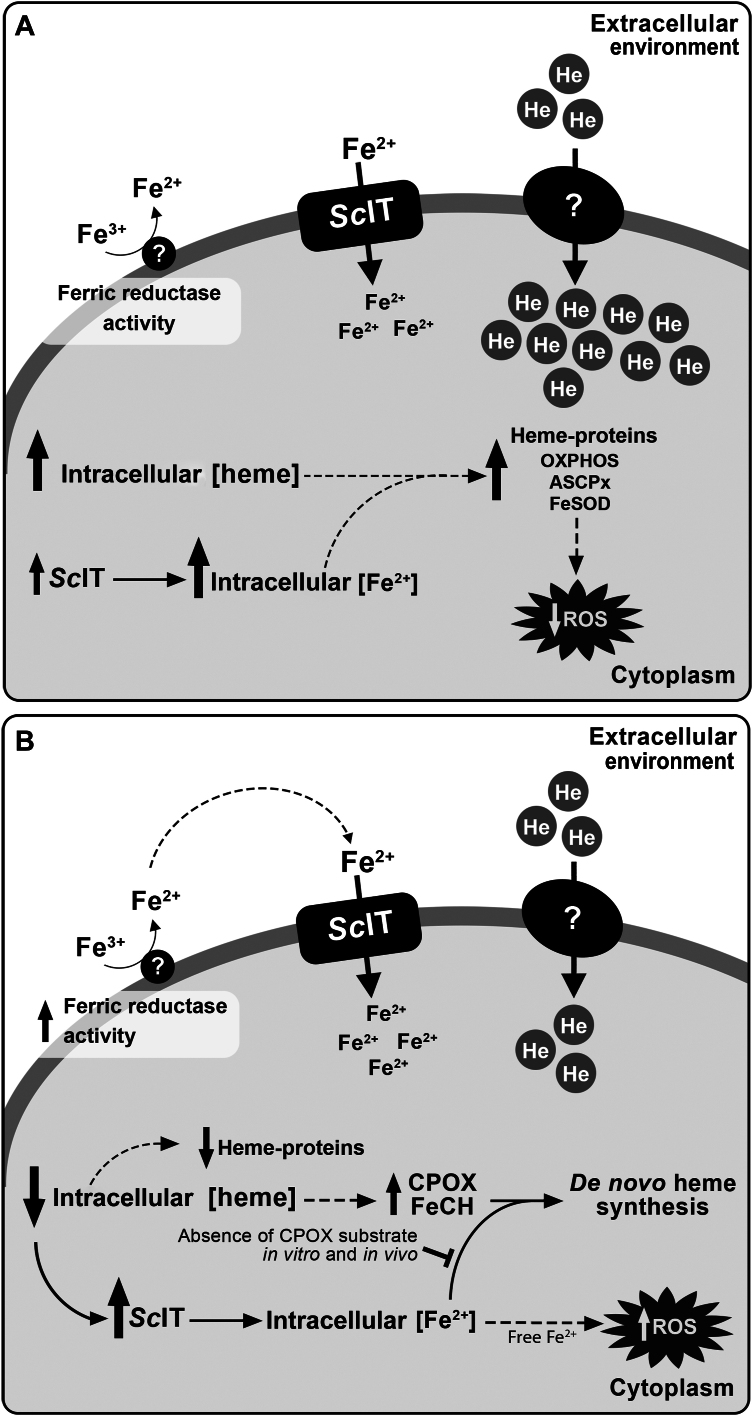
**Schematic representation of the main findings observed in WTR and Apo strains and the proposed hypotheses.***A*, in the WTR strain, the elevated intracellular heme concentration is maintained by an increase in heme uptake. Also, part of the intracellular Fe^2+^ detected in this protozoan is attributed to the elevated protein abundance and activity of *Sc*IT. As a consequence of the high intracellular heme and Fe^2+^ concentrations in the WTR strain, the synthesis and activity of heme-proteins, those found in OXPHOS and the antioxidant metabolism (ASCPx and FeSOD), would be favored, resulting in a consequent reduction in ROS production. *B*, in the Apo strain, the low intracellular heme concentration results from reduced uptake and symbiont elimination. The label-free PRM-MS data show an increase in the abundance of CPOX and FeCH in this protozoan, suggesting a mechanism for *de novo* heme synthesis to deal with decreased heme availability. Similarly, the abundance of *Sc*IT is favored by the low intracellular heme concentration and supports its *de novo* synthesis. However, in the Apo strain, the substrate of CPOX is not found *in vitro* or *in vivo*, preventing the *de novo* heme synthesis. Thus, the free Fe^2+^ taken up by *Sc*IT would lead to excessive ROS production, and the low heme concentration would impair the synthesis and activity of heme-proteins.

In conclusion, the study of *S. culicis* unveiled intricate mechanisms governing heme synthesis and uptake in response to H_2_O_2_ resistance induction and symbiont elimination, highlighting their potential ramifications on cellular processes. Also, the label-free PRM-MS approach significantly advanced our understanding of the heme biosynthetic pathway in these protozoa. Figure 13 summarizes the main findings observed here and presents the proposed hypotheses. Compared to WT, the WTR strain exhibited increased heme uptake, resulting in a higher intracellular concentration. Although further analysis is required to confirm which heme transporter is present in *S. culicis*, it has been shown that such transporter can uptake iron-protoporphyrin through an active process that requires the presence of Na^+^ and/or K^+^ ions. Additionally, WTR exhibited a high intracellular Fe^2+^ concentration when compared to WT. Notably, despite the elevated abundance and activity of *Sc*IT in WTR, only a portion of the Fe^2+^ content is attributed to it. At the same time, direct ferrous iron transport by *Sc*IT in the WT strain would be very low or nonexistent, suggesting the presence of other means for Fe^2+^ acquisition in *S. culicis*. The high intracellular heme and Fe^2+^ concentrations in the WTR strain would favor the synthesis and activity of heme-proteins, those found in OXPHOS and the antioxidant metabolism (ASCPx and FeSOD), leading to a consequent reduction in ROS production (Fig. 13*A*). In the Apo strain, the low intracellular heme concentration, in comparison to WT, results from reduced uptake and symbiont elimination. The label-free PRM-MS data showed an increase in the abundance of CPOX and FeCH in the Apo strain, suggesting a mechanism for *de novo* heme synthesis to deal with decreased heme availability and symbiont elimination. Similarly, the abundance of *Sc*IT, the primary Fe^2+^ transporter in Apo, is favored by the low intracellular heme concentration and supports its *de novo* synthesis. However, in the Apo strain, the substrate of CPOX is not found *in vitro* or *in vivo* in the midgut of the invertebrate host, hampering the *de novo* heme synthesis by the protozoan and its growth in nature. Thus, the free Fe^2+^ taken up by *Sc*IT would lead to excessive ROS production *via* Fenton reaction, and the low heme concentration would impair the synthesis and activity of heme proteins in the Apo strain (Fig. 13*B*).

## Experimental procedures

### Protozoa culture and growth conditions

*S. culicis* WT (COLPROT041) and Apo (COLPROT034) strains were provided by Fiocruz Protist Collection (http://colprot.fiocruz.br), whereas the development of the WTR strain was performed as previously described (23). Epimastigotes (extracellular insect-stage protozoa) from the three strains were maintained at 28 °C in SM, which was prepared according to Sadigursky and Brodskyn (26) with modifications. Briefly, basic LIT media [5 g L^−1^ liver infusion broth (BD Difco), 5 g L^−1^ tryptose (Sigma-Aldrich), 68.5 mM NaCl, 5.4 mM KCl, 56.4 mM Na_2_HPO_4_, and 0.2% (w/v) D-glucose] was supplemented with 10% FBS (Cultilab) and 15 μM bovine hemin (Sigma-Aldrich). Epimastigotes of three-day-old culture (log phase) were used for all experiments, and except where noted below, analyses were performed with protozoa grown in SM. Alternatively, epimastigotes were grown in HDM, which was composed of LIT media supplemented only with 10% heme-depleted FBS. Heme-depleted FBS was obtained after filtration of FBS over Amicon Ultra-15 10,000 NMWL columns (Merck Millipore) to remove low molecular mass components (39). Heme depletion was verified by measuring the absorbance at 405 nm using a SpectraMax M3 Multi-Mode Microplate Reader (Molecular Devices) (37).

### Quantification of the enzymatic components from the heme biosynthetic pathway by label-free PRM-MS

Protein extraction and sample preparation: Epimastigotes (10^8^ cells/ml) were washed thrice in 10 mM PBS (pH 7.2) and resuspended in 80 μl of sample lysis solution [50 mM Tris–HCl pH 7.6 and 2% (w/v) SDS] plus protease inhibitor cocktail with EDTA (Roche Applied Science). Ten freeze-thaw cycles in liquid nitrogen were conducted, followed by a 5 min boiling and centrifugation at 16,000 *g* for 10 min; the supernatants were stored at −80 °C until use. After that, protein concentration was determined using the 2D Quant kit (GE Healthcare, Buckinghamshire) according to the manufacturer's instructions. One hundred micrograms of protein from each lysate supernatant were processed according to the filter-aided sample preparation protocol (61), using only porcine trypsin (Promega) for protein enzymatic digestion. The final peptide solution was concentrated under vacuum centrifugation to approximately 50 μl, and TFA was added to a final concentration of 1%. Peptides were then desalted Poros R2 (Thermo Fisher Scientific) homemade tip columns, dried under vacuum centrifugation, and stored at −20 °C until use.

Mass spectrometry analysis: After peptide desalting, samples were resuspended in 80 μl of 1% formic acid and quantified using the Pierce Quantitative Colorimetric Peptide Assay kit (Thermo Fisher Scientific). For each sample, 10 μl containing 1 μg of desalted tryptic peptide digest and 60 fmol Pierce Peptide Retention Time Calibration Mixture (Thermo Fisher Scientific) were injected into a 2-cm long (100 μm internal diameter) trap column packed with 3 μm ReproSil-Pur 120 Å C18-AQ matrix (Dr Maisch GmbH), followed by separation on a 200 cm μPAC C18 (2.5 μm of interpillar distance) (PharmaFluidics) column connected to a 20 μm emitter (10 μm of internal diameter) (New Objective). Chromatography was carried out on the EASY-nLC 1200 system (Thermo Fisher Scientific). Chromatographic separation occurred at 50 °C and 400 nl/min, using 0.1% (v/v) formic acid in water and 0.1% (v/v) formic acid in 80% (v/v) acetonitrile as mobile phases A and B, respectively. For untargeted DDA analysis, peptides were separated on a 0 to 50% B gradient over 160 min, while for the label-free PRM-MS analysis, the separation occurred by a 0 to 30% B gradient over 120 min, followed by a 30 to 45% B gradient over 40 min. The eluted peptides were directly introduced into a Q Exactive Plus Orbitrap mass spectrometer (Thermo Fisher Scientific) for analysis. The source voltage was set to 1.9 kV, the capillary temperature to 250 °C, and the tube lens voltage 60 V. For DDA analysis, MS1 spectra (300–1500 *m/z*) were acquired in profile mode at 70,000 resolution (for *m/z* = 200), automatic gain control (AGC) target of 1e6, and maximum injection time (max. IT) of 100 ms. For each spectrum, the 12 most intense ions (isolation window and offset of 2.0–0.5 *m/z*, respectively) were submitted to higher energy collisional dissociation fragmentation (normalized collision energy of 30 units), followed by MS2 acquisition (200–2000 *m/z*) in centroid mode at 17,500 resolution, AGC target of 5e4, and max. IT of 50 ms. The dynamic exclusion option was selected for a duration of 30 s, and precursors with *z* = 1 or unassigned were not submitted to fragmentation. For label-free PRM-MS, MS1 spectra (300–1500 *m/z*) were acquired in profile mode at 35,000 resolution, AGC of 1e6, and max. IT of 110 ms. Peptide ions with a pre-specified *m/z* and eluted in a retention time window, previously determined by the calculator tool in Skyline software (version 21.1.0.278; https://skyline.ms/project/home/begin.view) (62, 63) (Table S2), were selected (isolation window and offset of 1.0–0.5 *m/z*, respectively) and submitted to higher energy collisional dissociation fragmentation as described above, followed by MS2 acquisition (fixed first mass at 100 *m/z*) in profile mode at 17,500 resolution, AGC target of 2e5, and max. IT of 100 ms.

Proteomics data analysis: Peptide-spectrum matching was performed using the Comet search engine (version 2016.01), which is available as part of the computational environment of PatternLab for Proteomics (version 4.0, http://patternlabforproteomics.org) (64). Searches were performed against sequences for *S. culicis* and *Candidatus* Kinetoplastibacterium blastocrithidii, downloaded from the DDBJ/EMBL/GenBank [PRJNA170971 and CP003733 (25), respectively]. The final search database, constructed by the PatternLab search database generator tool, included a reverse decoy for each target sequence, sequences from common contaminants, and sequences from *Saccharomyces cerevisiae* corresponding to Pierce Peptide Retention Time Calibration Mixture. The following parameters were used: semi-tryptic peptide candidates with a mass between 550 and 5500 Da; peptide sequences with up to two missed cleavages; 40 ppm for the precursor mass; methionine oxidation as variable modification and carbamidomethyl cysteine as fixed modification. The statistical validation was performed by the Search Engine Processor (SEPro) (65). Identifications obtained in the DDA analysis were used to build a spectral library loaded to Skyline software (62, 63). The following parameters were used in Skyline's transition settings: peptides with precursor charges two or three and ion types y and b with charges one or 2; precursor exclusion window of 0.05 *m/z*; ion match tolerance of 0.05 *m/z* and 0.055 *m/z* relative to spectral library and instrument, respectively. Also, only tryptic peptides with 7 to 25 amino acids and spectral library annotation were evaluated (Table S2). The AUC_norm_ was obtained for each peptide ion by dividing the area under the curve and the total ion current of the same technical replicate. Next, this value was multiplied by the median total ion current for all replicate analyses for all strains.

### Determination of intracellular heme and Fe^2+^ concentrations

Epimastigotes (10^7^ cells/ml) were grown for 0, 24, 48, and 72 h at 28 °C, counted in a Neubauer chamber, washed thrice with PBS, and kept dry at −20 °C until use. Protein homogenates were prepared by sonication, as previously described (66). Briefly, the pellet was resuspended in cold PBS (2.5 × 10^8^ cells/ml) containing protease inhibitor cocktail (Sigma-Aldrich) and disrupted on ice by sonication for 10 cycles of 7 s with intervals of 7 s, using a Markson GE50 Ultrasonic Processor. Amplitude was set to 70%, and cell disruption was monitored by light microscopy. Analysis of intracellular heme concentration was performed by the pyridine method. Briefly, 400 μl cells homogenate was diluted in pyridine solution [20% (v/v) NaOH 1 M, 48% (v/v) pyridine (Sigma-Aldrich), and 32% (v/v) ultrapure water] and an oxidized spectrum (500–600 nm) was recorded in Shimadzu UV-2550 spectrophotometer (Shimadzu). Next, diluted samples were reduced with sodium dithionite (Sigma-Aldrich), and a new spectrum was acquired. The intracellular heme concentration was determined through the difference between oxidized and reduced spectra, considering absorbance values at 557 nm and 541 nm and using the extinction coefficient of 23.98 mM^−1^ cm^−1^ (67). Intracellular Fe^2+^ concentration was evaluated using a commercial kit (Bioclin K017–1 kit), following the manufacturer's protocol with some modifications. Briefly, 200 μl cells homogenate was added to 95 μl reduction buffer, and absorbance was recorded at 550 nm using SpectraMax M3 Multi-Mode Microplate Reader. After that, 5 μl of 12 mM ferrozine solution was added, and samples were incubated for 30 min at 37 °C, followed by another absorbance measurement at 550 nm. The concentration of Fe^2+^ was determined using a standard curve with known FeSO_4_ concentrations (1–10 μg/ml). Alternatively, intracellular heme and Fe^2+^ concentrations were determined in epimastigotes grown for 72 h at 28 °C in (a) SM with addition of 1 mM FeSO_4_, 50 μM DIP (Sigma-Aldrich), or 50 μM DIP + 1 mM FeSO_4_; (b) HDM alone or with the addition of 10 μM hemin or 10 μM PPIX (Frontier Scientific).

### Determination of heme analog uptake by flow cytometry

Epimastigotes (10^7^ cells/ml) were washed thrice with PBS and incubated in MES buffer [10 mM MES (Sigma-Aldrich), 140 mM NaCl, 5 mM KCl, 1 mM CaCl_2_, and 0.09% (w/v) D-glucose, pH 7.4] for 2 h at 28 °C. At this point, 10 μM Zn-PPIX (Frontier Scientific) was added to the buffer and incubated for 15 min at 28 °C. After incubation with Zn-PPIX, protozoa were centrifuged and washed for 5 min with ice-cold 5% bovine serum albumin (BSA) in PBS to remove porphyrin nonspecifically bound to the plasma membrane. To determine whether Zn-PPIX uptake through the plasma membrane is carried out by an active transport process, protozoa were pre-incubated in MES buffer with 5 μM Omy (Sigma-Aldrich), 2 μM AA (Sigma-Aldrich), and 1 mM KCN (Merck Millipore) for 30 min at 28 °C. To analyze the involvement of secondary active transporters in Zn-PPIX uptake, protozoa were incubated with 50 μM CCCP (Sigma-Aldrich), 100 μM valinomycin (Sigma-Aldrich), and 1 μM ionomycin (Sigma-Aldrich) in combination with Zn-PPIX (excitation at 405 nm and emission 660 nm) (41). A total of 10,000 events were acquired in the region previously established to correspond to protozoa in a CytoFLEX Flow Cytometer (Beckman Coulter) equipped with the CytExpert software (Beckman Coulter). Data analysis was performed using CytExpert 2.5 software.

### *In silico* analysis

BLAST homology searches of *S. culicis* genome [available through DDBJ/EMBL/GenBank database under the accession no. PRJNA170971 (25)] identified homologous sequences to *L*IT1 (16), *Tc*IT (17), IRT1 (18), *L*HR1 (37), *Tc*HRG (40, 68), *Tb*HRG (39), and *L*FLVCRb (38) (available from the TriTryp and GeneBank database under the accession no. LmjF.31.3070, TCDM_06386, AAB01678.1, LmjF.24.2230, TcCLB.504037.10 and TcCLB.511071.190, Tb927.8.6010, and LmjF.17.1430, respectively). Sequences with the highest identity and similarity values were selected, analyzed for protein structure with PHYRE (www.sbg.bio.ic.ac.uk/phyre/) (69), and visualized with the standard molecular viewer PyMOL (version 2.5, https://pymol.org/). Multiple amino acid sequence alignments were obtained by BioEdit Sequence Alignment Editor (version 7.2.6.1, https://bioedit.software.informer.com/) using the ClustalW alignment tool (69, 70).

### qPCR analysis

Epimastigotes (10^8^ cells/ml) were washed thrice with PBS, resuspended in 1 ml TRIzol reagent (Thermo Fisher Scientific), and processed according to the manufacturer's instructions for RNA extraction. Total RNA was quantified in a spectrophotometer, and cDNA synthesis was performed with a SuperScriptVilo kit (Thermo Fisher Scientific). All primer pairs (Table S3) were tested by conventional PCR using a 35-cycle reaction (denaturation at 95 °C for 30 s, annealing at 60 °C for 30 s, and extension at 72 °C for 30 s) and analyzed in 2% agarose gel. The PCR reaction products were purified using Illustra GFX PCR DNA and Gel Band Purification Kit (GE Healthcare) and sequenced with BigDye Terminator v3.1 Cycle Sequencing Kit (Thermo Fisher Scientific) in 3730 DNA Analyzer (Applied Biosystems). qPCR was performed in an ABI Prism 7500 FAST (Applied Biosystem) using Go-Taq PCR Master Mix (Promega). The Comparative 2^-ΔΔCT^ method was used to compare changes in gene expression levels (71). Genes from actin and 69 kDa paraflagellar rod protein (Table S3) were used as endogenous controls. Alternatively, gene expression analysis was performed in epimastigotes grown for 72 h at 28 °C in (a) SM with the addition of 1 mM FeSO_4_, 50 μM DIP, or 50 μM DIP + 1 mM FeSO_4_; (b) HDM alone or with addition of 10 μM hemin or 10 μM PPIX.

### Immunogold labeling for SEM

Epimastigotes (5.0 × 10^7^ cells/ml) were washed with PBS and incubated with primary rabbit anti-*Sc*IT polyclonal antibody (dilution 1:50) (Rhea Biotech) or pre-immune rabbit IgG (dilution 1:50) (Rhea Biotech) for 1 h at 28 °C in MES buffer supplemented with 10% FBS. After that, cells were washed thrice and fixed with 2% paraformaldehyde (Sigma-Aldrich) for 40 min at room temperature. Cells were washed thrice, maintained in blocking buffer (10% FBS and 50 mM ammonium chloride in PBS) for 30 min, and incubated with secondary goat anti-rabbit IgG-gold 10 nm antibody (dilution 1:75) (Sigma-Aldrich) diluted in blocking buffer for 1 h at room temperature. Cells were fixed again with 2.5% glutaraldehyde (Sigma-Aldrich) diluted in 0.1 M Na-cacodylate buffer (pH 7.2) for 40 min and post-fixed with 1% osmium tetroxide (Sigma-Aldrich) for 20 min, both at room temperature. After the washings, dehydration was performed in ethanol (50%, 70%, 90%, and 100%). Samples were dried by the critical point method with CO_2_, mounted on aluminum stubs, and sputter-coated with carbon to observe the cell surface in detail (72). Samples were observed in a Field Emission Scanning Electron Microscope Magellan 400L (FEI Company) and in a Focused Ion Beam Dual Beam Helios NanoLab 650 Microscope (FEI Company). All images were obtained *via* backscattered electron, using a concentric back scattered detector, at an accelerating voltage of 18 kV.

To assess the specificity of primary rabbit anti-*Sc*IT polyclonal antibody, indirect ELISA and dot blot analyses were conducted. The ELISA was performed using the synthetic peptide (MGQSVSETTSLCEGLQGDYS) at a concentration of 1.0 μg/ml, followed by incubation of anti-*Sc*IT at the following serial dilutions: 1:1000, 1:2000, 1:4000, 1:8000, 1:16000, 1:32000, and 1:64000. Detection was achieved using a secondary HRP-labeled donkey anti-rabbit IgG (Bioleged) and H_2_O_2_/OPD was used as the chromogenic substrate. The absorbance was read at 492 nm. Dot blot was performed applying 1.0, 0.5, 0.25, and 0.12 μg of the synthetic peptide, and the membranes were incubated with TBS-Tween buffer (20 mM Tris, 150 mM NaCl, 0.1% Tween-20, pH7.5) + 2% bovine serum albumin for 1h at room temperature. After that, anti-*Sc*IT polyclonal antibody (dilution 1:1000) or pre-immune rabbit IgG (dilution 1:1000) were added using the same buffer, and membranes were incubated overnight, followed by detection with the secondary HRP-labeled antibody.

### Detection of *Sc*IT by flow cytometry

Epimastigotes (10^7^ cells/ml) were washed thrice with PBS and fixed in 2% paraformaldehyde for 40 min at room temperature. Cells were washed, maintained in blocking buffer for 30 min, and incubated with primary anti-*Sc*IT rabbit polyclonal antibody (dilution 1:125) or pre-immune rabbit IgG (dilution 1:125) diluted in blocking buffer for 1 h at room temperature. Cells were washed thrice and incubated with secondary goat anti-rabbit Alexa Fluor 488 antibody (dilution 1:2000) (Invitrogen) for 1 h at room temperature. A total of 10,000 events were acquired in the region previously established to correspond to protozoa using either a FACSCalibur Flow Cytometer (Becton Dickinson) equipped with Cell Quest software (Joseph Trotter, Scripps Research Institute) or a CytoFLEX Flow Cytometer equipped with CytExpert software. Data analysis was performed using Summit 6.1 software (Beckman Coulter) or CytExpert 2.5 software. Alternatively, *Sc*IT abundance was determined in epimastigotes grown for 72 h at 28 °C in HDM alone or with the addition of 10 μM hemin or 10 μM PPIX.

### Determination of ferric iron reductase activity

Epimastigotes (10^8^ cells/ml) were washed thrice with PBS and incubated in MES buffer containing 1 mM K_3_[Fe(CN)]_6_ and 500 μM NADPH (Sigma-Aldrich) for 2 h at 28 °C. After that, cells were centrifuged at 10,000*g* for 5 min, and the supernatant (200 μl) was monitored at 420 nm using a SpectraMax M3 Multi-Mode Microplate Reader to evaluate the reduction of the ferric into the ferrous form of K_3_[Fe(CN)_6_]. The concentration of the ferric form of K_3_[Fe(CN)_6_] was determined using a standard curve with known concentrations (1–1000 μM) (73). Alternatively, K_3_[Fe(CN)_6_] reduction was determined in epimastigotes grown for 72 h at 28 °C in HDM alone or with the addition of 10 μM hemin or 10 μM PPIX.

### Determination of ferrous iron uptake

Epimastigotes (10^8^ cells/ml) were washed thrice with PBS and incubated with anti-*Sc*IT rabbit polyclonal antibody (dilution 1:50) or pre-immune rabbit IgG (dilution 1:50) diluted in MES buffer supplemented with 10% FBS for 1 h at 28 °C. After that, 1 mM FeSO_4_ and 50 μM ascorbic acid (Sigma-Aldrich) were added, and cells were incubated for 1 h at 28 °C. Cells were washed thrice and processed to determine intracellular Fe^2+^ concentration. Briefly, 200 μl cells homogenate was added to 95 μl reduction buffer, and absorbance was recorded at 550 nm using SpectraMax M3 Multi-Mode Microplate Reader. After that, 5 μl of 12 mM ferrozine solution was added, and samples were incubated for 30 min at 37 °C, followed by another absorbance measurement at 550 nm. The concentration of Fe^2+^ was determined using a standard curve with known FeSO_4_ concentrations (1–10 μg/ml).

### Statistical analysis

All data were analyzed using Prism software (version 8.0.1; GraphPad) and expressed as the mean ± SD or mean ± SEM. Data distribution was evaluated by the Shapiro–Wilk normality test. Means were compared by one-way ANOVA or Kruskal–Wallis test associated with Dunn's, Dunnett's, or Tukey's post-test, as indicated in the text or the figure legends. Differences were considered statistically significant when *p* ≤ 0.05.

## Data availability

Mass spectrometry data were deposited to the ProteomeXchange Consortium *via* the PanoramaPublic repository with the dataset identifier PXD044748.

## Supporting information

This article contains supporting information (10, 25).

## Conflict of interest

The authors declare that they have no conflicts of interest with the contents of this article.

## References

[1] Kostygov A.Y., Karnkowska A., Votýpka J., Tashyreva D., Maciszewski K., Yurchenko V. (2021). Euglenozoa: taxonomy, diversity and ecology, symbioses and viruses. Open Biol..

[2] Teixeira M.M.G., Borghesan T.C., Ferreira R.C., Santos M.A., Takata C.S.A., Campaner M. (2011). Phylogenetic validation of the genera *Angomonas* and *Strigomonas* of Trypanosomatids harboring bacterial endosymbionts with the description of new species of trypanosomatids and of proteobacterial symbionts. Protist.

[3] Votýpka J., Kostygov A.Y., Kraeva N., Grybchuk-Ieremenko A., Tesařová M., Grybchuk D. (2014). *Kentomonas* gen. n., a new genus of endosymbiont-containing trypanosomatids of Strigomonadinae subfam. n. Protist.

[4] Archibald J.M. (2015). Endosymbiosis and eukaryotic cell evolution. Curr. Biol..

[5] Brum F.L., Catta-Preta C.M.C., De Souza W., Schenkman S., Elias M.C., Motta M.C. (2014). Structural characterization of the cell division cycle in *Strigomonas culicis*, an endosymbiont-bearing trypanosomatid. Microsc. Microanal..

[6] Catta-Preta C.M.C., Brum F.L., da Silva C.C., Zuma A.A., Elias M.C., de Souza W. (2015). Endosymbiosis in trypanosomatid protozoa: the bacterium division is controlled during the host cell cycle. Front. Microbiol..

[7] Motta M.C., Catta-Preta C.M., Schenkman S., de Azevedo Martins A.C., Miranda K., de Souza W. (2010). The bacterium endosymbiont of *Crithidia deanei* undergoes coordinated division with the host cell nucleus. PLoS One.

[8] Alves J.M.P.P., Klein C.C., Da Silva F.M., Costa-Martins A.G., Serrano M.G., Buck G.A. (2013). Endosymbiosis in trypanosomatids: the genomic cooperation between bacterium and host in the synthesis of essential amino acids is heavily influenced by multiple horizontal gene transfers. BMC Evol. Biol..

[9] Klein C.C., Alves J.M.P., Serrano M.G., Buck G.A., Vasconcelos A.T.R., Sagot M.F. (2013). Biosynthesis of vitamins and cofactors in bacterium-harbouring trypanosomatids depends on the symbiotic association as revealed by genomic analyses. PLoS One.

[10] Alves J.M.P., Voegtly L., Matveyev A.V., Lara A.M., da Silva F.M., Serrano M.G. (2011). Identification and phylogenetic analysis of heme synthesis genes in trypanosomatids and their bacterial endosymbionts. PLoS One.

[11] Furuyama K., Kaneko K., Vargas P.D. (2007). Heme as a magnificient molecule with multiple missions: heme determines its own fate and governs cellular homeostasis. Tohoku J. Exp. Med..

[12] Kořený L., Lukeš J., Oborník M. (2010). Evolution of the haem synthetic pathway in kinetoplastid flagellates: an essential pathway that is not essential after all?. Int. J. Parasitol..

[13] Chang K.P., Trager W. (1974). Nutritional significance of symbiotic bacteria in two species of hemoflagellates. Science.

[14] Chang K.P., Chang C.S., Sassa S. (1975). Heme biosynthesis in bacterium protozoon symbioses: enzymic defects in host hemoflagellates and complemental role of their intracellular symbiotes. Proc. Natl. Acad. Sci. U. S. A..

[15] Fenton H.J.H. (1894). Lxxiii. - oxidation of tartaric acid in presence of iron. J. Chem. Soc. Trans..

[16] Huynh C., Sacks D.L., Andrews N.W. (2006). A *Leishmania amazonensis* ZIP family iron transporter is essential for parasite replication within macrophage phagolysosomes. J. Exp. Med..

[17] Dick C.F., Rocco-Machado N., Dos-Santos A.L.A., Carvalho-Kelly L.F., Alcantara C.L., Cunha-E-Silva N.L. (2022). An iron transporter is involved in iron homeostasis, energy metabolism, oxidative stress, and metacyclogenesis in *Trypanosoma cruzi*. Front. Cell Infect. Microbiol..

[18] Eide D., Broderius M., Fett J., Guerinot M.L. (1996). A novel iron-regulated metal transporter from plants identified by functional expression in yeast. Proc. Natl. Acad. Sci. U. S. A..

[19] Eng B.H., Guerinot M.L., Eide D., Saier M.H. (1998). Sequence analyses and phylogenetic characterization of the ZIP family of metal ion transport proteins. J. Membr. Biol..

[20] Guerinot M.L. (2000). The ZIP family of metal transporters. Biochim. Biophys. Acta - Biomembr..

[21] Mittra B., Cortez M., Haydock A., Ramasamy G., Myler P.J., Andrews N.W. (2013). Iron uptake controls the generation of *Leishmania* infective forms through regulation of ROS levels. J. Exp. Med..

[22] Kořený L., Oborník M., Lukeš J. (2013). Make it, take it, or leave it: heme metabolism of parasites. PLoS Pathog..

[23] Bombaça A.C.S., Dias F.A., Ennes-Vidal V., Garcia-Gomes A., dos S., Sorgine M.H.F. (2017). Hydrogen peroxide resistance in *Strigomonas culicis*: effects on mitochondrial functionality and *Aedes aegypti* interaction. Free Radic. Biol. Med..

[24] Bombaça A.C.S., Brunoro G.V.F., Dias-Lopes G., Ennes-Vidal V., Carvalho P.C., Perales J. (2020). Glycolytic profile shift and antioxidant triggering in symbiont-free and H_2_O_2_-resistant *Strigomonas culicis*. Free Radic. Biol. Med..

[25] Motta M.C.M., Martins A.C., de Souza S.S.A., Catta-Preta C.M.C., Silva R., Klein C.C. (2013). Predicting the proteins of *Angomonas deanei, Strigomonas culicis* and their respective endosymbionts reveals new aspects of the Trypanosomatidae family. PLoS One.

[26] Sadigursky M., Brodskyn C.I. (1986). A new liquid medium without blood and serum for culture of hemoflagellates. Am. J. Trop. Med. Hyg..

[27] Renberg R.L., Yuan X., Samuel T.K., Miguel D.C., Hamza I., Andrews N.W. (2015). The heme transport capacity of *L*HR1 determines the extent of virulence in *Leishmania amazonensis*. PLoS Negl. Trop. Dis..

[28] Constable E.C., Housecroft C.E. (2019). The early years of 2,2′-Bipyridine—a ligand in its own lifetime. Molecules.

[29] Jacques I., Andrews N.W., Huynh C. (2010). Functional characterization of *L*IT1, the *Leishmania amazonensis* ferrous iron transporter. Mol. Biochem. Parasitol..

[30] D’avila-Levy C.M., Boucinha C., Kostygov A., Santos H.L.C., Morelli K.A., Grybchuk-Ieremenko A. (2015). Exploring the environmental diversity of kinetoplastid flagellates in the high-throughput DNA sequencing era. Mem. Inst. Oswaldo Cruz..

[31] Maslov D.A., Opperdoes F.R., Kostygov A.Y., Hashimi H., Lukeš J., Yurchenko V. (2019). Recent advances in trypanosomatid research: genome organization, expression, metabolism, taxonomy and evolution. Parasitology.

[32] Chang K. (1975). Reduced growth of *Blastocrithidia culicis* and *Crithidia oncopelti* freed of intracellular symbiotes by chloramphenicol. J. Protozool..

[33] Cuervo P., Domont G.B., De Jesus J.B. (2010). Proteomics of trypanosomatids of human medical importance. J. Proteomics.

[34] van Bentum M., Selbach M. (2021). An Introduction to advanced targeted acquisition methods. Mol. Cell Proteomics.

[35] Brunoro G.V.F., Menna-Barreto R.F.S., Garcia-Gomes A.S., Boucinha C., Lima D.B., Carvalho P.C. (2019). Quantitative proteomic map of the trypanosomatid *Strigomonas culicis*: the biological contribution of its endosymbiotic bacterium. Protist.

[36] Orrego L.M., Cabello-Donayre M., Vargas P., Martínez-García M., Sánchez C., Pineda-Molina E. (2019). Heme synthesis through the life cycle of the heme auxotrophic parasite *Leishmania major*. FASEB J..

[37] Huynh C., Yuan X., Miguel D.C., Renberg R.L., Protchenko O., Philpott C.C. (2012). Heme uptake by *Leishmania amazonensis* is mediated by the transmembrane protein *L*HR1. PLoS Pathog..

[38] Cabello-Donayre M., Orrego L.M., Herráez E., Vargas P., Martínez-García M., Campos-Salinas J. (2020). *Leishmania* heme uptake involves *Lm*FLVCRb, a novel porphyrin transporter essential for the parasite. Cell Mol. Life Sci..

[39] Cabello-Donayre M., Malagarie-Cazenave S., Campos-Salinas J., Gálvez F.J., Rodríguez-Martínez A., Pineda-Molina E. (2016). Trypanosomatid parasites rescue heme from endocytosed hemoglobin through lysosomal HRG transporters. Mol. Microbiol..

[40] Merli M.L., Pagura L., Hernández J., Barisón M.J., Pral E.M.F., Silber A.M. (2016). The *Trypanosoma cruzi* protein *Tc*HTE is critical for heme uptake. PLoS Negl. Trop. Dis..

[41] Cabello-Donayre M., Orrego L.M., Herráez E., García-Hernández R., Pérez-Victoria J.M. (2022). New insights on heme uptake in *Leishmania* spp. Int. J. Mol. Sci..

[42] Tenhunen R., Marver H.S., Schmid R. (1968). The enzymatic conversion of heme to bilirubin by microsomal heme oxygenase. Proc. Natl. Acad. Sci. U. S. A..

[43] Lechuga G.C., Pereira M.C.S., Bourguignon S.C. (2019). Heme metabolism as a therapeutic target against protozoan parasites. J. Drug Target..

[44] Cupello M.P., Souza C.F., Menna-Barreto R.F., Nogueira N.P.A., Laranja G.A.T., Sabino K.C.C. (2014). Trypanosomatid essential metabolic pathway: new approaches about heme fate in *Trypanosoma cruzi*. Biochem. Biophys. Res. Commun..

[45] El-Sayed N.M., Myler P.J., Bartholomeu D.C., Nilsson D., Aggarwal G., Tran A.N. (2005). The genome sequence of *Trypanosoma cruzi*, etiologic agent of Chagas disease. Science.

[46] Okada K. (2009). The novel heme oxygenase-like protein from *Plasmodium falciparum* converts heme to bilirubin IXα in the apicoplast. FEBS Lett..

[47] Sigala P.A., Crowley J.R., Hsieh S., Henderson J.P., Goldberg D.E. (2012). Direct tests of enzymatic heme degradation by the malaria parasite *Plasmodium falciparum*. J. Biol. Chem..

[48] Taylor M.C., Kelly J.M. (2010). Iron metabolism in trypanosomatids, and its crucial role in infection. Parasitology.

[49] Schell D., Borowy N.K., Overath P. (1991). Transferrin is a growth factor for the bloodstream form of *Trypanosoma brucei*. Parasitol. Res..

[50] Lima M.F., Villalta F. (1990). *Trypanosoma cruzi* receptors for human transferrin and their role. Mol. Biochem. Parasitol..

[51] Porto-Carreiro I., Attias M., Miranda K., De Souza W., Cunha-E-Silva N. (2000). *Trypanosoma cruzi* epimastigote endocytic pathway: cargo enters the cytostome and passes through an early endosomal network before storage in reservosomes. Eur. J. Cell Biol..

[52] Sardar A.H., Kumar S., Kumar A., Purkait B., Das S., Sen A. (2013). Proteome changes associated with *Leishmania donovani* promastigote adaptation to oxidative and nitrosative stresses. J. Proteomics.

[53] Pinho N., Bombaça A.C., Wiśniewski J.R., Dias-Lopes G., Saboia-Vahia L., Cupolillo E. (2022). Nitric oxide resistance in *Leishmania (Viannia) braziliensis* involves regulation of glucose consumption, glutathione metabolism and abundance of pentose phosphate pathway enzymes. Antioxidants (Basel, Switzerland).

[54] Xiang L., Laranjeira-Silva M.F., Maeda F.Y., Hauzel J., Andrews N.W., Mittra B. (2019). Ascorbate-dependent peroxidase (APX) from *Leishmania amazonensis* is a reactive oxygen species-induced essential enzyme that regulates virulence. Infect. Immun..

[55] Bombaça A.C.S., Gandara A.C.P., Ennes-Vidal V., Bottino-Rojas V., Dias F.A., Farnesi L.C. (2021). *Aedes aegypti* infection with trypanosomatid *Strigomonas culicis* alters midgut redox metabolism and reduces mosquito reproductive fitness. Front. Cell Infect. Microbiol..

[56] Tripodi K.E.J., Menendez Bravo S.M., Cricco J.A. (2011). Role of heme and heme-proteins in trypanosomatid essential metabolic pathways. Enzyme Res..

[57] Azevedo-Martins A.C., Machado A.C.L., Klein C.C., Ciapina L., Gonzaga L., Vasconcelos A.T.R. (2015). Mitochondrial respiration and genomic analysis provide insight into the influence of the symbiotic bacterium on host trypanosomatid oxygen consumption. Parasitology.

[58] Kořený L., Sobotka R., Kovářová J., Gnipová A., Flegontov P., Horváth A. (2012). Aerobic kinetoplastid flagellate *Phytomonas* does not require heme for viability. Proc. Natl. Acad. Sci. U. S. A..

[59] Chaumont F., Schanck A.N., Blum J.J., Opperdoes F.R. (1994). Aerobic and anaerobic glucose metabolism of *Phytomonas* sp. isolated from *Euphorbia characias*. Mol. Biochem. Parasitol..

[60] Silva F.M., Kostygov A.Y., Spodareva V.V., Butenko A., Tossou R., Lukeš J. (2018). The reduced genome of *Candidatus* Kinetoplastibacterium sorsogonicusi, the endosymbiont of *Kentomonas sorsogonicus* (Trypanosomatidae): loss of the haem-synthesis pathway. Parasitology.

[61] Wiśniewski J.R., Zougman A., Nagaraj N., Mann M. (2009). Universal sample preparation method for proteome analysis. Nat. Methods.

[62] Pino L.K., Searle B.C., Bollinger J.G., Nunn B., MacLean B., MacCoss M.J. (2020). The Skyline ecosystem: informatics for quantitative mass spectrometry proteomics. Mass Spectrom. Rev..

[63] MacLean B., Tomazela D.M., Shulman N., Chambers M., Finney G.L., Frewen B. (2010). Skyline: an open source document editor for creating and analyzing targeted proteomics experiments. Bioinformatics.

[64] Carvalho P.C., Lima D.B., Leprevost F.V., Santos M.D.M., Fischer J.S.G., Aquino P.F. (2016). Integrated analysis of shotgun proteomic data with PatternLab for proteomics 4.0. Nat. Protoc..

[65] Carvalho P.C., Fischer J.S., Xu T., Cociorva D., Balbuena T.S., Valente R.H. (2012). Search engine processor: filtering and organizing peptide spectrum matches. Proteomics.

[66] Menna-Barreto R.F., Goncalves R.L., Costa E.M., Silva R.S., Pinto A.V., Oliveira M.F. (2009). The effects on *Trypanosoma cruzi* of novel synthetic naphthoquinones are mediated by mitochondrial dysfunction. Free Radic. Biol. Med..

[67] Berry E.A., Trumpower B.L. (1987). Simultaneous determination of hemes a, b, and c from pyridine hemochrome spectra. Anal. Biochem..

[68] Tevere E., Di Capua C.B., Chasen N.M., Etheridge R.D., Cricco J.A. (2023). *Trypanosoma cruzi* heme responsive gene (*Tc*HRG) plays a central role in orchestrating heme uptake in epimastigotes. FEBS J..

[69] Kelley L.A., Sternberg M.J.E. (2009). Protein structure prediction on the web: a case study using the phyre server. Nat. Protoc..

[70] Hall T.A. (1999). BIOEDIT: a user-friendly biological sequence alignment editor and analysis program for Windows 95/98/NT. Nucleic Acids Symp. Ser..

[71] Livak K.J., Schmittgen T.D. (2001). Analysis of relative gene expression data using real-time quantitative PCR and the 2-ΔΔCT method. Methods.

[72] Tenaglia A.H., Luján L.A., Ríos D.N., Molina C.R., Midlej V., Iribarren P.A. (2023). Antibodies to variable surface antigens induce antigenic variation in the intestinal parasite *Giardia lamblia*. Nat. Commun..

[73] Dick C.F., de Moura Guimarães L., Carvalho-Kelly L.F., Cortes A.L., da Silva Lara Morcillo L., da Silva Sampaio L. (2020). A ferric reductase of *Trypanosoma cruzi* (*Tc*FR) is involved in iron metabolism in the parasite. Exp. Parasitol..

