# Supplementary material for: Heme metabolism in Strigomonas culicis: Implications of H2O2 resistance induction and symbiont elimination
Source: J Biol Chem. 2024 Aug 17;300(9):107692. doi: 10.1016/j.jbc.2024.107692 (PMC11648444; doi:10.1016/j.jbc.2024.107692)
Supplement: Supporting information [file mmc1.docx]

Heme metabolism in *Strigomonas culicis*: Implications of H_2_O_2_ resistance induction and symbiont elimination

Ana Cristina Souza Bombaça^1,2^, Marcelle Almeida Caminha^1,3^, Juliana Magalhães Chaves Barbosa^1^, Yasmin Pedra-Rezende^1,3^, Vitor Ennes-Vidal^2^, Giselle Villa Flor Brunoro^4^, Bráulio Soares Archanjo^5^, Claudia Masini d’Avila^2^, Richard Hemmi Valente^3^, Rubem Figueiredo Sadok Menna-Barreto^2,^*

^1^Laboratório de Biologia Celular, Instituto Oswaldo Cruz, Fundação Oswaldo Cruz, Rio de Janeiro 21040-900, Brazil;

^2^Laboratório de Doenças Parasitárias, Instituto Oswaldo Cruz, Fundação Oswaldo Cruz, Rio de Janeiro 21040-900, Brazil;

^3^Laboratório de Toxinologia, Instituto Oswaldo Cruz, Fundação Oswaldo Cruz, Rio de Janeiro 21040-900, Brazil;

^4^Blau Farmacêutica S/A, São Paulo 06705-030, Brazil;

^5^Divisão de Metrologia de Materiais, Instituto Nacional de Metrologia, Qualidade e Tecnologia (Inmetro), Duque de Caxias 25250-020, Brazil.

*Correspondence: [rubemsadok@gmail.com/rubemb@ioc.fiocruz.br](mailto:rubemsadok@gmail.com/rubemb@ioc.fiocruz.br)

**Table S1:** Peptides identified by the data-dependent acquisition (DDA) analysis. The peptides whose precursor ions were used to build a spectral library in Skyline Software and selected for label-free parallel reaction monitoring targeted mass spectrometry (PRM-MS) analyses are in boldface.

| **Protein** | **UniProtKB** | **Peptide sequences^a^** |
| --- | --- | --- |
| *S. culicis*_GltX | S9VN50 | AIEDDLTTLGVSYDSGPTFSSDYFR |
|  |  | **LQLTDAEQGK** |
|  |  | **SDVVFLDAEDVALIK** |
|  |  | VYPTYDF**CC**PIIDSVEGVTHALR |
|  |  | FPPEASGFLHIGHAK |
|  |  | NHNFILNTTNK |
| *Ca.* K. blastocrithidii_GltX | G1C9N4 | NYEPNENDLGSIIDLFK |
|  |  | STTLNQLAENAIIFYK |
|  |  | FAPSPTGFLHLGGAR |
|  |  | IYLTGEANTPSINK |
| HemA | G1C9N0 | **DNIPSILELINK** |
|  |  | NIVSEAVILST**C**NR |
|  |  | NHISISSASIDLVK |
|  |  | NITLYSIDDLQK |
|  |  | TVFQELEDLSVLFIGAGEIIELSIK |
|  |  | VDPSYFYNYVNDIAVK |
| GSA | G1C9M6 | DTIVLEFNNIESVEK |
|  |  | FEG**C**YHGHSDSLLVK |
|  |  | AGSGLLTLGQPTSSGVPTSFIK |
|  |  | **AQQFIPGGVNSPVR** |
|  |  | **NTNLLTSELQR** |
|  |  | SAQGLFGISPDITTLAK |
| ALAD | G1C9M2 | LESNEYK |
|  |  | **C**IDLGIPTIALFPVIDSTLK |
|  |  | EAAADITEGADMLIVKPGIPYLDVLTK |
|  |  | **NSYQINPSNINEAIR** |
|  |  | **QSLLHASAGVDIVAPSDMMDGR** |
|  |  | **TNNGIESINANGLIPR** |
|  |  | VPTFAYQVSGEYSMIK |
|  |  | YSSAFYGPFR |
| PBGD | G1C9L8 | SLYPEIVVDFLVIVTQGDK |
|  |  | VLGGS**C**QVPLAANAYIK |
|  |  | ISSYIDPSISLPAAGQGAIGIQIR |
|  |  | LASAQAEHVR |
|  |  | SLVSSLS**C**IDTTF**C**VK |
|  |  | **SVYSISIK** |
| UROS | G1C9L4 | FIVTHQR |
|  |  | IKPFDLK |
|  |  | ILSDSLNDAGFETLSLPSLK |
|  |  | IYNFLSSYFIK |
|  |  | VSEY**C**EVDL**C**SVYFR |
| UROD | G1C9L0 | **GGSLWLK** |
|  |  | LVNNELK |
|  |  | VPLIGFSGSPWTLA**C**YMVEGQSSK |
| CPOX | G1C9R7 | **EACEPFGADVYPR** |
|  |  | **GGVNFSHVFGK** |
|  |  | **GTQFGLQSGGR** |
|  |  | **NPHVPTSHANVR** |
|  |  | VIADGDVIEK |
|  |  | YAEFNLVIDR |
| HemN | G1C9K6 | **C**PY**C**DFNSHSIDPNNIDEELFLK |
|  |  | NFIYDLK |
|  |  | QDALNSITK |
|  |  | SFNPNHLSIYHLTIEPNTFFSK |
|  |  | SIISVFIGGGTPSILSVESIDK |
| PPOX | G1C9N8 | ELHQFVR |
|  |  | **LTGGDTDPTTEK** |
|  |  | LVLGASVIYGNFAK |
|  |  | **VICEALVEAIR** |
|  |  | **YLVLYSTK** |
|  |  | **AAVFAGALYYPR** |
| *S. culicis*_FeCH | G1C9Q4 | **FAATGDDYPEQCYETTR** |
|  |  | **SASCGTSPLSLYTYSIAAK** |
|  |  | LGEHFH**C**GK |
|  |  | T**C**FVVSPSFSVD**C**LETLEEIAIATR |
| *Ca.* K. blastocrithidii_FeCH | G1C9K2 | ILIIPLYPQYSSSTTGSAIR |
|  |  | NAINSLVHNK |
|  |  | FATDYLTR |
|  |  | QLFIGAGGK |
| *Sc*IT | S9TKT7 | DPTDNVNR |
|  |  | DVPHGHSHAFEMPDDK |
|  |  | **LFSAPTIHPLHMR** |

^a^Peptides were selected from genomic sequences of the heme biosynthesis pathway previously described (10, 25).

**Table S2:** Peptides monitored by label-free parallel reaction monitoring targeted mass spectrometry (PRM-MS) with their pre-specified *m/z* and retention time window values.

| **Protein** | **UniProtKB** | **Peptide sequences^a^** | ***m/z*** | **Retention time window (min)** | |
| --- | --- | --- | --- | --- | --- |
|  |  |  |  | **Start** | **End** |
| *S. culicis*_GltX | S9VN50 | LQLTDAEQGK | 551.7906 | 66.64 | 74.64 |
|  |  | SDVVFLDAEDVALIK | 817.4378 | 148.62 | 154.62 |
| HemA | G1C9N0 | DNIPSILELINK | 684.8903 | 156.08 | 162.08 |
| GSA | G1C9M6 | NTNLLTSELQR | 644.8464 | 101.20 | 109.20 |
|  |  | AQQFIPGGVNSPVR | 735.3966 | 102.56 | 106.56 |
| ALAD | G1C9M2 | NSYQINPSNINEAIR | 866.9343 | 108.70 | 112.70 |
|  |  | TNNGIESINANGLIPR | 841.9446 | 121.67 | 127.67 |
|  |  | QSLLHASAGVDIVAPSDMMDGR | 757.3683 | 130.99 | 136.99 |
| PBGD | G1C9L8 | SVYSISIK | 448.7580 | 91.56 | 95.56 |
| UROD | G1C9L4 | GGSLWLK | 380.7212 | 89.04 | 97.04 |
| CPOX | G1C9R7 | NPHVPTSHANVR | 443.5655 | 44.82 | 50.82 |
|  |  | GTQFGLQSGGR | 554.2807 | 67.48 | 75.48 |
|  |  | GGVNFSHVFGK | 383.5331 | 100.98 | 104.98 |
|  |  | EACEPFGADVYPR | 755.8352 | 102.99 | 106.99 |
| PPOX | G1C9N8 | LTGGDTDPTTEK | 617.7935 | 45.35 | 51.35 |
|  |  | YLVLYSTK | 493.7815 | 96.00 | 100.00 |
|  |  | AAVFAGALYYPR | 649.8482 | 122.63 | 128.63 |
|  |  | VICEALVEAIR | 636.8527 | 123.69 | 129.69 |
| *S. culicis*_FeCH | G1C9Q4 | FAATGDDYPEQCYETTR | 1012.421 | 85.96 | 89.96 |
| *Sc*IT | S9TKT7 | LFSAPTIHPLHMR | 507.2783 | 110.20 | 118.20 |
| Pierce™ Peptide Retention Time Calibration Mixture |  | SSAAPPPPPR | 493.7683 | 50.52 | 54.52 |
|  |  | GISNEGQNASIK | 613.3168 | 51.80 | 57.80 |
|  |  | HVLTSIGEK | 496.2867 | 58.39 | 64.39 |
|  |  | IGDYAGIK | 422.7364 | 67.74 | 73.74 |
|  |  | TASEFDSAIAQDK | 695.8324 | 79.15 | 85.15 |
|  |  | SAAGAFGPELSR | 586.8003 | 86.37 | 92.37 |
|  |  | ELGQSGVDTYLQTK | 773.8956 | 95.64 | 101.64 |
|  |  | GLILVGGYGTR | 558.3260 | 112.68 | 118.68 |
|  |  | GILFVGSGVSGGEEGAR | 801.4115 | 115.74 | 121.74 |

^a^Peptides were selected from genomic sequences of the heme biosynthetic pathway previously described (10, 25).

**Table S3:** Oligonucleotide sequences used in real-time qPCR assays.

| **Gene** | **GenBank accession number** | **Primers sequence (5’-3’)^a^** | **E-value^b^** |
| --- | --- | --- | --- |
| *Sc*IT | ATMH01009756.1 | TGTGCAGGGGGTGGATCGTC *For*  TGCGCGGGCATTCTGCTGTA *Rev* | 1e^-20^ |
| UROS | JF756609.1 | TCCGATAGTCTTAACGATGCTGG *For*  TCCCTGGAAAGGGTACATCC *Rev* | 1e^-29^ |
| CPOX | ATMH01001800.1 | TCCTCATGTCGCTGCCTCCC *For*  CATTCAGCCAGTCGCGGTCCT *Rev* | 5e^-36^ |
| Actina | ATMH01000150.1 | CGGGAGAACGTGGAGCGACA *For*  GTATGGGTTTCGCCGTCCA *Rev* | 2e^-14^ |
| 69 kDa Paraflagellar rod protein | ATMH01001313.1 | CGGGAGAACGTGGAGCGACA *For*  TTCGATGCGGCGCTTGACCT *Rev* | 6e^-25^ |

^a^Sequences were designed from *S. culicis* and *Ca*. Ki. Blastocrithidii genome assigned as PRJNA170971 and CP003733 in GenBank (25).

^b^Identity of amplified transcripts.

**Figure S1: Uroporphyrinogen III synthase (UROS) and coproporphyrinogen III oxidase (CPOX) transcripts levels.** Gene expression analysis of UROS and coproporphyrinogen III oxidase CPOX in WTR and Apo strains. Data were normalized using the transcripts levels of WT strain (dashed black line). Actin and 69 kDa paraflagellar rod protein were used as endogenous controls. Data are represented as mean ± SEM of at least five independent experiments. Significant p-values were obtained by one-way ANOVA test followed by Dunnett's post-test (***p ≤ 0.01).

**Figure S2:** ***Sc*HT and *Sc*FLVCRa alignments.** Alignment of amino acid sequences of **(A)** *Sc*HT from *S. culicis*, *L*HR1 from *L. major*, *Tc*HRG from *T. cruzi*, and *Tb*HRG from *T.* *brucei* or **(B)** *Sc*FLVCRa from *S. culicis* and *L*FLVCRb from *L. major.* Identical residues are highlighted in black, and similar residues in gray. Predicted transmembrane domains are underlined in black, while conserved residues shown to be critical for the heme transport function of *L*HR1 are boxed in red. **(C)** Table showing the percentage of identity (gray) and similarity (white) of the amino acid sequences between *L*FLVCRb, *Sc*FLVCRa, and other FLVCR proteins identified in the *S. culicis* database.

**Figure S3: Specificity analysis of primary rabbit anti-*Sc*IT polyclonal antibody.** **(A)** Indirect ELISA was performed using the synthetic peptide (MGQSVSETTSLCEGLQGDYS) at a concentration of 1.0 µg/ml, followed by incubation with primary rabbit anti-*Sc*IT polyclonal antibody at the following serial dilutions: 1:1,000, 1:2,000, 1:4,000, 1:8,000, 1:16,000, 1:32,000, and 1:64,000. Detection was achieved using a secondary HRP-labeled donkey anti-rabbit IgG. **(B)** Dot blot was performed applying 1.0, 0.5, 0.25, and 0.12 µg of the synthetic peptide, following incubation of the membranes with anti-*Sc*IT antibody (dilution 1:1,000) or pre-immune rabbit IgG (dilution 1:1,000) overnight. Detection was also performed with a secondary HRP-labeled donkey anti-rabbit IgG.

**Figure S4: Ultrastructural analysis of *S. culicis* labeled with anti-*Sc*IT antibody.** The presence of *Sc*IT was analyzed on the surface of the three *S. culicis* strains, combining scanning electron microscopy (SEM) with immunogold labeling. **(A, B)** Micrographs of secondary goat anti-rabbit IgG-gold 10 nm antibody obtained by scanning transmission electron microscopy (STEM). Analysis of WT strain **(C, D)** without any labeling, **(E, F)** labeled only with secondary goat anti-rabbit IgG-gold 10 nm antibody (dilution: 1:50), or **(G, H)** with pre-immune rabbit IgG (dilution 1:50) instead of primary polyclonal antibody and secondary goat anti-rabbit IgG-gold 10 nm antibody (dilution: 1:50). **(D, F, H)** Micrographs of the areas enclosed by the white rectangle were enlarged to visualize the gold particles better. White arrowheads indicate isolated gold particles on the protozoa surface. All images were obtained via backscattered electron (BSE), using a concentric back scattered (CBS) detector, at an accelerating voltage of 18 kV. Bars = 1 µm **(C, E, G)**, 500 nm **(A),** and 100 nm **(B, D, F, H)**.
